# Electronic Patient-Reported Outcome System Implementation in Outpatient Cardiovascular Care: A Randomized Clinical Trial

**DOI:** 10.1001/jamanetworkopen.2024.54084

**Published:** 2025-01-14

**Authors:** Shuhei Yamashita, Yoshinori Katsumata, Shun Kohsaka, Hiroki Kitakata, Yasuyuki Shiraishi, Koki Yamaoka, Yuki Muramoto, Tomohiko Ono, Satoshi Shoji, Keishiro Yagyu, Yasushi Oginosawa, Masaharu Kataoka, Masahiro Hashimoto, Shigeru B. H. Ko, Yuko Kitagawa, Masahiro Jinzaki

**Affiliations:** 1 Department of Cardiology, Keio University School of Medicine, Tokyo, Japan; 2 Institute for Integrated Sports Medicine, Keio University School of Medicine, Tokyo, Japan; 3 Department of Cardiology, National Hospital Organization Saitama Hospital, Saitama, Japan; 4 Duke Clinical Research Institute, Durham, North Carolina; 5 Department of Cardiology, Hino Municipal Hospital, Tokyo, Japan; 6 The Second Department of Internal Medicine, University of Occupational and Environmental Health, Kitakyushu, Japan; 7 Department of Radiology, Keio University School of Medicine, Tokyo, Japan; 8 Department of Systems Medicine, Keio University, Tokyo, Japan; 9 Department of Surgery, Keio University School of Medicine, Tokyo, Japan

## Abstract

**Importance:**

The integration of patient-reported outcome (PRO) assessments in cardiovascular care has encountered considerable obstacles despite their established clinical relevance.

**Objective:**

To assess the impact of a physician- and patient-friendly electronic PRO (ePRO) monitoring system on the quality of cardiovascular care in clinical practice.

**Design, Setting, and Participants:**

This open-label, multicenter, pilot randomized clinical trial was phase 2 of a multiphase study that was conducted from October 2022 to October 2023 and focused on the implementation and evaluation of an ePRO monitoring system in outpatient clinics in Japan. During phase 1 (conducted from December 2021 to March 2022), this ePRO monitoring system was developed at a single outpatient center using feedback from patients and physicians. Eligible patients were older than 18 years with a clinical diagnosis of heart failure (HF), atrial fibrillation (AF), or coronary artery disease (CAD). Participants were randomly assigned 1:1 to either the ePRO or control group. Statistical analysis was based on the intention-to-treat approach.

**Intervention:**

Patients in the ePRO group were asked to complete the 12-item Kansas City Cardiomyopathy Questionnaire (KCCQ-12), Atrial Fibrillation Effect on Quality-of-Life Questionnaire (AFEQT), or Seattle Angina Questionnaire (SAQ) on smart tablets before their clinical examination. Results were reviewed by physicians at each outpatient visit. The control group received usual care consisting of medication management, lifestyle modification, and invasive procedures in accordance with clinical guidelines. Both groups had 5 monthly outpatient visits.

**Main Outcomes and Measures:**

Patient satisfaction, quality of information (QOI) provided by physicians, and disease knowledge were assessed using questionnaires (eg, Patient Satisfaction Questionnaire [PSQ]; score range: 5-25, with higher scores indicating greater patient satisfaction) at baseline and the fifth visit, over approximately 4 months.

**Results:**

Of the 50 patients included, 48 (median [SD] age, 71.0 [62.3-75.0] years; 28 males [58.3%]) completed follow-up. The prevalence was 56.2% (n = 27) for HF, 75.0% (n = 36) for AF, and 10.4% (n = 5) for CAD. The distribution of PRO measures used was 21 patients (43.8%) for KCCQ-12, 24 (50.0%) for AFEQT, and 3 (6.2%) for SAQ. Compared with the control group, the ePRO group showed significant improvements in mean (SD) PSQ score (0.16 [2.06] vs 1.61 [1.75]; *P* = .01) and QOI score regarding treatment (−0.12 [0.53] vs 0.35 [0.71]; *P* = .01). The ePRO group exhibited a greater increase in the PSQ score regarding communication (−0.12 [0.53] vs 0.43 [0.90]; *P* = .01).

**Conclusions and Relevance:**

In this randomized clinical trial, implementation of the ePRO monitoring system significantly enhanced patient-physician communication and the clarity of physicians’ explanations about treatment. These findings suggest that the ePRO monitoring system is capable of supporting patient-centered cardiovascular care.

**Trial Registration:**

University Hospital Medical Information Network Identifier: UMIN000049251

## Key Points

**Question:**

Can physicians develop user-friendly electronic patient-reported outcome (ePRO) monitoring systems to improve the quality of care in cardiovascular practice?

**Findings:**

In this pilot randomized clinical trial of 50 patients in outpatient cardiovascular clinics, a physician-developed ePRO monitoring system significantly improved patient satisfaction, particularly with physician communication and clarity of treatment explanations compared with usual care.

**Meaning:**

These findings suggest that the ePRO monitoring system could support patient-centered care in cardiovascular practice.

## Teaser

This randomized clinical trial evaluates changes in patient satisfaction, quality of information provided by physicians, and patient disease knowledge among adults with a diagnosis of heart failure, atrial fibrillation, or coronary artery disease who received electronic patient-reported outcome monitoring.

## Introduction

Contemporary cardiovascular disease management aims not only to reduce adverse clinical events but also to enhance the quality of life and foster a shared understanding of disease management between patients and physicians. However, achieving satisfactory care has become increasingly challenging due to the increasing complexity of cardiovascular conditions, diverse management decisions, and heterogeneity of patient perspectives and values.^1,2^

Patient-reported outcomes (PROs) are essential metrics for optimizing care by quantifying patient symptoms, function, and quality of life.^3^ In routine clinical practice, PROs highlight substantial discrepancies in symptom recognition between patients and physicians.^4,5,6^ Underrecognition of patient symptoms can markedly affect physicians’ management decisions, particularly concerning invasive therapies.^7,8,9^ Additionally, PROs have prognostic significance in cardiovascular care^10,11,12^ and are now recommended by clinical guidelines for use in routine assessment of treatment success and enhancing patient care.^13,14,15^

Electronic PRO (ePRO) systems, such as those using online surveys, mobile applications, or automated telephone interfaces, have proven feasible for identifying symptoms that can be treated by clinicians.^16^ For instance, in patients with cancer, ePRO monitoring enhances physical function, improves symptom management, reduces hospitalizations, and improves overall survival in those receiving cancer treatment.^17,18,19^ However, clinical research in cardiovascular care has revealed more heterogeneous outcomes,^20,21^ highlighting the necessity for a more dedicated system to improve physician-patient communication.

To explore the optimal PRO implementation strategy in cardiovascular care, we initially developed an ePRO monitoring system using qualitative methods, marking phase 1 of the full study. Subsequently, in phase 2, we conducted an open-label, multicenter pilot randomized clinical trial to assess the impact of this physician- and patient-friendly ePRO monitoring system on the quality of cardiovascular care in clinical practice.

## Methods

Phase 1 (December 2021-March 2022) focused on the development of the ePRO monitoring system, whereas phase 2 (October 2022-October 2023) involved the implementation and evaluation of this system. The trial protocol was approved by the Keio University Hospital Ethics Committee and by the local institutional review board of the 4 participating institutions in Japan. The full trial protocol and statistical analysis plan are provided in Supplement 1. All participants in phase 1 provided verbal informed consent, whereas patients in phase 2 provided electronic informed consent. This trial was conducted in accordance with the Declaration of Helsinki^22^ and the Ethical Guidelines for Medical and Health Research Involving Human Subjects established by the Japanese Ministry of Health, Labour and Welfare. We followed the Consolidated Standards of Reporting Trials (CONSORT) reporting guideline.

### Development of the ePRO Monitoring System

In phase 1, an electronic survey system was developed based on feedback from patients and physicians (eFigure 1 in Supplement 2). The prototype was designed by core working members, including board-certified cardiologists (S.Y., Y.K., H.K., Y.S., and S.K.). It includes 3 reliable and validated disease-specific questionnaires: the 12-item Kansas City Cardiomyopathy Questionnaire (KCCQ-12; score range: 0-100, with the highest score indicating best health status), Atrial Fibrillation Effect on Quality-of-Life Questionnaire (AFEQT; score range: 0-100, with the highest score indicating best health status), and Seattle Angina Questionnaire (SAQ; score range: 0-100, with the highest score indicating best health status), which quantify the impact of heart failure (HF), atrial fibrillation (AF), and coronary artery disease (CAD), respectively, on patient symptoms, function, and quality of life.^23,24,25^ Next, we included 9 consecutive patients being followed up at the outpatient cardiology clinic at Keio University Hospital (eTable 1 in Supplement 2) between December 2021 and March 2022. These patients provided feedback on the ePRO monitoring system through open-ended questions about its usability and Likert-scale questionnaires focusing on screen visualization and survey methods. To refine the system for physicians unfamiliar with PROs, we obtained feedback on utility and screen visualization from 2 other board-certified cardiologists who do not routinely assess PROs in clinical care. Their comments were evaluated and used to revise the system.

### Patient and Physician Insights Into Use of the ePRO Monitoring System in Clinical Practice 

During phase 1, the questionnaire results showed minimal issues with visualization (size, font, and figure placement), and PRO surveys at every outpatient visit seemed preferable (eFigure 4 in Supplement 2). The open-ended feedback from patients recognized areas for improvement in visual presentation and limitations of the disease-specific questionnaires (eTable 4 in Supplement 2). Some patients indicated the importance of visualizing the trajectory of PRO scores: “It will be easier to understand if the changes in scores before and after are displayed.” Given the association between changes in KCCQ-12 scores and subsequent cardiovascular events,^26^ the PRO trajectory potentially provides information that is of interest to both physicians and patients. Furthermore, some patients noted the difficulty in interpreting the scores: “I want to know the meaning of the AFEQT scores. I cannot understand whether a higher or lower score is better.” Accordingly, we used a bar graph to display the PRO trajectory on a single screen and added a threshold to interpret AFEQT scores, in line with previous research.^11^ Some patients also noted limitations in these questionnaires: “I have a knee problem and cannot climb stairs; therefore, it is difficult to answer questions about climbing stairs.” After refining them based on patients’ assessments, 2 board-certified cardiologists provided feedback (eTable 5 in Supplement 2), and the final version was created (eFigures 5-7 in Supplement 2). The ePRO monitoring system has not yet been integrated into the electronic medical records.

### Implementation and Evaluation of the ePRO Monitoring System

In phase 2, we evaluated health status monitoring using the ePRO monitoring system refined in phase 1. Eligible patients were older than 18 years and diagnosed with HF, AF, or CAD. HF was defined as a brain natriuretic peptide level of 100 pg/mL or greater (to convert to nanogram per liter, multiply by 1.0) at enrollment, AF was identified by electrocardiography, and CAD was diagnosed based on previous coronary catheterization confirming angina or myocardial infarction. Patients who were hospitalized within 1 month or scheduled for elective hospitalization were excluded. Prior to randomization, patients with HF, AF, or CAD were assigned to the KCCQ-12, AFEQT, or SAQ groups, respectively. For patients with 2 or more of these 3 coexisting cardiovascular diseases, physicians assigned an appropriate PRO measure based on the primary cardiovascular disease.

**Figure 1.**
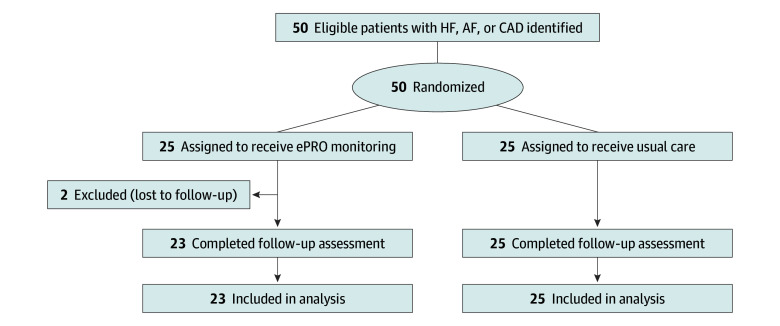
CONSORT Study Flow Diagram AF indicates atrial fibrillation; CAD, coronary artery disease; ePRO, electronic patient-reported outcome; and HF, heart failure.

Patients were randomized to either the ePRO or control group in a 1:1 ratio using a software-generated allocation sequence (Figure 1). Adaptive randomization ensured no more than a 2-patient difference in PRO assignment between groups. Based on the patients’ insights into the surveillance method, they were followed up a mean (SD) time of every 4 (2) weeks for 5 visits. The control group received standard care (medication management, lifestyle modification, and invasive procedures) in accordance with clinical practice guidelines, while the ePRO group completed assigned questionnaires (KCCQ-12, AFEQT, or SAQ) on smart tablets before their clinical examination. The health-related information from PROs was shared between physicians and patients at every outpatient visit (eFigure 2 in Supplement 2).

### Outcomes

The primary outcomes were patient satisfaction, quality of information (QOI), and disease knowledge. Patient satisfaction was evaluated at baseline and at the final follow-up using 5 items from the Patient Satisfaction Questionnaire (PSQ) Short Form (eTable 2 in Supplement 2).^27^ Each adopted question was rated using a 5-point Likert scale. The 5-item PSQ score ranged from 5 to 25, with higher scores indicating greater patient satisfaction. The PSQ Short Form contains 18 questions assessing several subscales of satisfaction with medical care. After extensive discussions, we identified 4 subscales—communication, technical quality, interpersonal manner, and time spent with doctor—that could be influenced by ePRO monitoring. From these subscales, we selected 5 questions suitable for the Japanese outpatient research setting. Patients rated their overall satisfaction using a Likert scale ranging from 1 (most dissatisfied) to 10 (most satisfied).

QOI disclosure by physicians was measured using 2 items of the well-established, self-administered Prognosis and Treatment Perception Questionnaire (PTPQ). Although traditionally used in cancer research and focused on patients’ perspectives,^28^ the PTPQ was modified for cardiovascular care.^29^ Patient perception of the QOI regarding treatment and prognosis at baseline and at the final follow-up was assessed using a 5-point Likert scale (5: excellent, 4: good, 3: satisfactory, 2: fair, and 1: poor) (eTable 2 in Supplement 2).

To investigate the effect of ePRO monitoring on disease knowledge, patients with HF or AF answered multiple-choice questions about their disease (eTable 3 in Supplement 2). The trajectory of HF and its effect on life expectancy were evaluated in patients with HF. Specifically, patients were asked to choose the most typical HF trajectory from 4 diagrams representing different illness trajectories (gradual, intermittent, rapid, or temporal decline), based on previous studies^29^ (eFigure 3 in Supplement 2). The first 3 HF trajectories were based on a palliative care review,^30^ and the fourth, temporal decline, was subsequently added. Questions about the impact of HF on life expectancy were adapted from a previous study.^31^ Patients rated their perspectives on HF as follows: (1) will be cured completely, (2) will last for the rest of their lives without shortening their life expectancy, (3) will shorten their life expectancy, and (4) unclear on how HF would affect their lives. Furthermore, these patients answered 8 questions on AF therapy, symptom recognition, and attitudes; these questions were derived from previous studies.^32,33^ Patients’ disease knowledge levels were then calculated by summing the number of correct answers. Disease knowledge scores ranged from 0 to 2 points for HF and from 0 to 8 points for AF. HF knowledge was categorized as excellent (2 points), good (1 point), or poor (0 points), whereas AF knowledge was classified as excellent (6-8 points), good (3-5 points), or poor (0-2 points).

All patients completed the questionnaires at their initial and final visits. Changes in patient satisfaction, QOI, and disease knowledge scores were also assessed. Power calculations were not performed due to the pilot study design.

### Statistical Analysis

Because phase 2 was a pilot trial, formal sample size calculations were not performed. In phase 1, we conducted an exploratory qualitative evaluation by collecting and examining narrative data from participants. Baseline characteristics were presented as medians with IQRs for continuous variables and as percentages for categorical variables. The mean (SD) changes in patient satisfaction and QOI regarding treatment and prognosis from baseline to the final follow-up were compared between the 2 groups using unpaired, 2-tailed *t* tests. Statistical testing was 2-sided, with *P* < .05 defined as statistically significant. All data were analyzed according to the intention-to-treat approach using R, version 4.6.0 (R Project for Statistical Computing).

## Results

### Baseline Characteristics of Patients in the Pilot Trial

Between March 2022 and June 2023, 50 patients were randomly assigned to receive PRO monitoring using an electronic system or usual care. Two patients were excluded for missing the follow-up visit; thus, 48 patients (25 in the control group; 23 in the ePRO group) were analyzed (Figure 1). The baseline characteristics of patients in the 2 groups were balanced, including 20 females (41.7%) and 28 males (58.3%), with a median (IQR) age of 71.0 (62.3-75.0) years (Table). The study population had a substantial burden of CV risk factors, including 29 patients (60.4%) with hypertension, 14 (29.2%) with diabetes, and 18 (37.5%) with dyslipidemia. The prevalence was 56.2% (n = 27) for HF, 75.0% (n = 36) for AF, and 10.4% (n = 5) for CAD. The distribution of physician-assigned PRO measures was 21 patients (43.8%) for KCCQ-12, 24 (50.0%) for AFEQT, and 3 (6.2%) for SAQ. All patients in both arms completed 5 scheduled visits, and all patients in the ePRO group successfully completed all ePRO surveys using a smart tablet. All patients completed questionnaires for outcome assessment at baseline and the final follow-up. Furthermore, all patients in the ePRO group completed disease-specific questionnaires, and the PROs were effectively collected.

**Table.** Patient Characteristics

Characteristics	Patients, No. (%)		
	Control group (n = 25)	ePRO group (n = 23)	Overall (n = 48)
Age, median (IQR), y	72.0 (65.0-75.0)	69.0 (60.5-75.5)	71.0 (62.3-75.0)
Sex			
Female	10 (40.0)	10 (43.5)	20 (41.7)
Male	15 (60.0)	13 (56.5)	28 (58.3)
BMI, median (IQR)	22.6 (21.1-24.8)	24.0 (20.7-26.5)	23.0 (20.9-26.5)
Comorbidities			
Heart failure	14 (56.0)	13 (56.5)	27 (56.2)
Atrial fibrillation	20 (80.0)	16 (69.6)	36 (75.0)
CAD	1 (4.0)	4 (17.4)	5 (10.4)
Hypertension	16 (64.0)	13 (56.5)	29 (60.4)
Diabetes	8 (32.0)	6 (26.1)	14 (29.2)
Dyslipidemia	7 (28.0)	11 (47.8)	18 (37.5)
CKD^a^	15 (60.0)	15 (65.2)	30 (62.5)
Stroke	3 (12.0)	1 (4.3)	4 (8.3)
Cancer	3 (12.0)	3 (13.0)	6 (12.5)
Echocardiographic parameters and laboratory tests			
LVEF, median (IQR), %	60.2 (45.0-66.0)	59.8 (43.3-69.6)	60.0 (44.0-66.6)
Left atrial diameter, median (IQR), mm	41.5 (32.8-45.5)	40.0 (36.0-44.0)	41.0 (35.0-45.0)
eGFR, median (IQR), mL/min/1.73 m^2^	56.9 (45.7-66.9)	54.9 (43.5-62.2)	55.0 (44.2-65.0)
BNP, median (IQR), pg/mL^b^	84.7 (42.2-143.5)	90.8 (44.0-170.7)	88.0 (40.9-160.3)
NT-proBNP, median (IQR), pg/mL^b^	800 (224-1397)	421 (186-1022)	559 (151-1253)
Physician-assigned PROs			
KCCQ-12	10 (40.0)	11 (47.8)	21 (43.8)
AFEQT	14 (56.0)	10 (43.5)	24 (50.0)
SAQ	1 (4.0)	2 (8.7)	3 (6.2)
Interval between each visit, wk	4.6 (4.3-5.0)	4.5 (4.5-4.9)	4.6 (4.3-5.0)

Abbreviations: AFEQT, Atrial Fibrillation Effect on Quality-of-Life Questionnaire (score range: 0-100, with the highest score indicating best health status); BMI, body mass index (calculated as weight in kilograms divided by height in meters squared); BNP, B-type natriuretic peptide; CAD, coronary artery disease; CKD, chronic kidney disease; eGFR, estimated glomerular filtration rate; KCCQ-12, 12-item Kansas City Cardiomyopathy Questionnaire (score range: 0-100, with the highest score indicating best health status); LVEF, left ventricular ejection fraction; NT-proBNP, N-terminal pro–B-type natriuretic peptide; PRO, patient-reported outcome; SAQ, Seattle Angina Questionnaire (score range: 0-100, with the highest score indicating best health status).

SI conversion factors: To convert BNP to nanogram per liter, multiply by 1.0.

^a^ CKD was defined as eGFR less than 60 mL/min/1.73 m^2^, calculated based on the formula eGFR = 194 × serum creatinine (mg/dL)^-1.094^ × age^-0.287^ × 0.739 (if female).

^b^ BNP and NT-proBNP values were missing in 12 patients (25.0%) and 36 patients (75.0%) among the entire population, respectively. Both values were missing in 3 patients (6.7%).

### Patient Satisfaction

**Figure 2.**
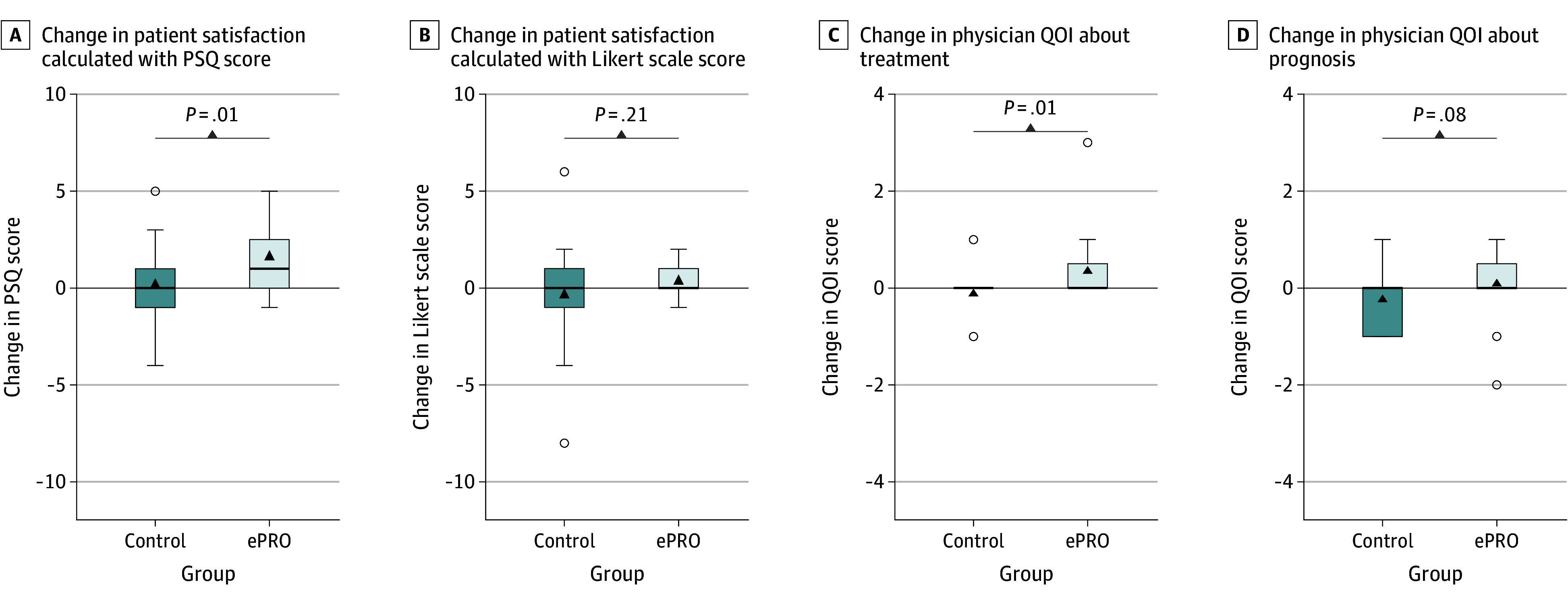
Changes in Patient Satisfaction and Quality of Information (QOI) Provided by Physician Upper and lower ends of the box represent the 25th to 75th percentiles of the IQR, with the horizontal line inside indicating the median; whiskers represent 1.5 times the IQR; black triangle represents the mean; and circles above or below boxes represent outliers. ePRO indicates electronic patient-reported outcome; PSQ, Patient Satisfaction Questionnaire.

Patient satisfaction expressed with mean (SD) PSQ scores (22.00 [2.38] and 20.65 [2.39]) and Likert scale scores (8.52 [1.53] and 8.65 [1.64]) at baseline was equal among patients in the control and ePRO groups (eTable 6 in Supplement 2). Compared with the control group, the mean (SD) PSQ score in the ePRO group significantly improved from baseline to the final follow-up (absolute change, 0.16 [2.06] vs 1.61 [1.75]; *P* = .01) (Figure 2A). Among the items, the mean (SD) score of question 1 regarding communication was significantly increased in the ePRO group (−0.12 [0.53] vs 0.43 [0.90]; *P* = .01). In contrast, the mean (SD) scores of questions in the other domains did not differ between the groups (eTable 7 in Supplement 2). Changes in patient satisfaction assessed using the Likert scale were not significantly different between the groups (Figure 2B).

### Quality of Information

Subsequently, we investigated whether ePRO monitoring in clinical practice improves the QOI of physicians. The ePRO group compared with the control group showed significantly greater improvement in mean (SD) scores for QOI about treatment (0.35 [0.71] vs −0.12 [0.53]; *P* = .01) (Figure 2C). Changes in mean (SD) scores for QOI about prognosis were not significantly different between the 2 groups (Figure 2D).

### Disease Knowledge

**Figure 3.**
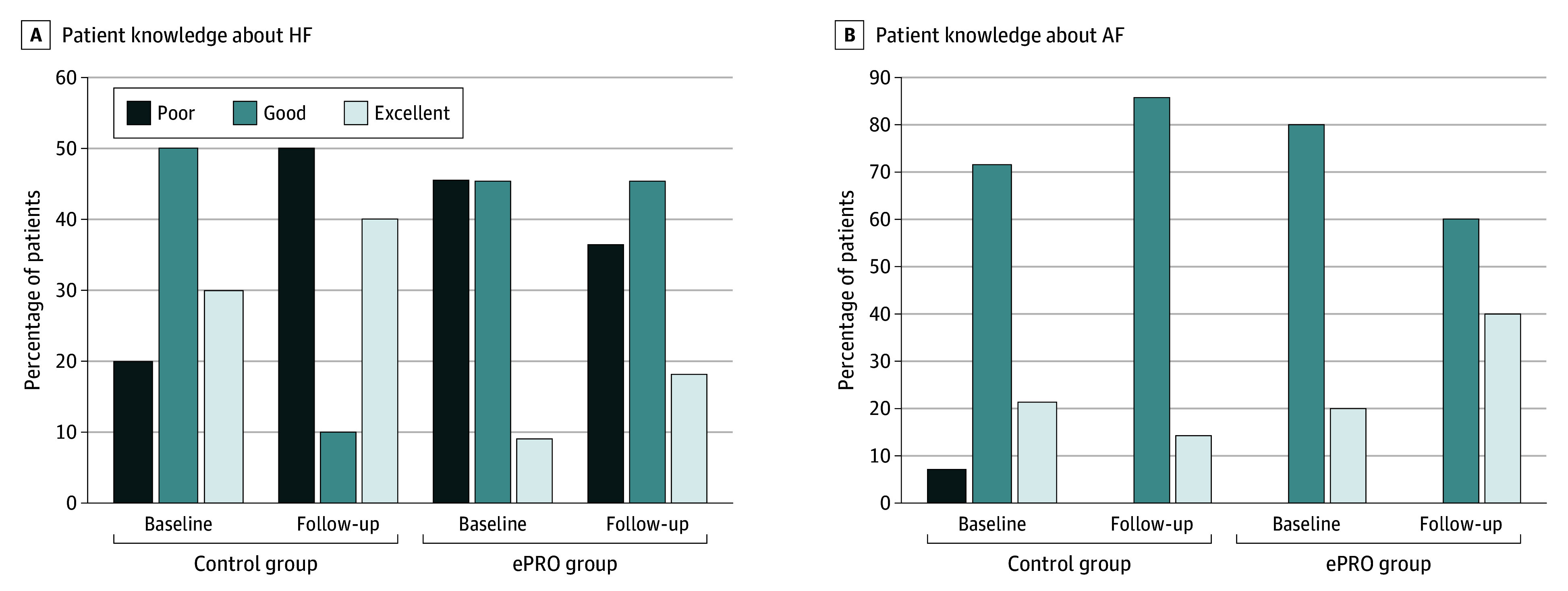
Patient Knowledge at Baseline and Final Follow-Up AF indicates atrial fibrillation; ePRO, electronic patient-reported outcome; and HF, heart failure.

Disease knowledge of HF in the ePRO group showed a favorable change compared with that in the control group (Figure 3A). While the percentage of patients classified with poor knowledge substantially increased from 20.0% (n = 2) to 50.0% (n = 5) in the control group of 10 patients, it decreased from 45.5% (n = 5) to 36.4% (n = 4) in the ePRO group of 11 patients. Similarly, a favorable change in disease knowledge regarding AF was observed in the ePRO group compared with the control group (Figure 3B). No patients had poor knowledge in either group at the follow-up assessment. The percentage of patients classified as having excellent knowledge partially decreased from 21.4% (n = 3) to 14.3% (n = 2) in the control group of 14 patients but increased from 20.0% (n = 2) to 40.0% (n = 4) in the ePRO group of 10 patients.

## Discussion

In this pilot trial integrating PRO monitoring into outpatient cardiovascular care, the ePRO monitoring system, developed with extensive multidisciplinary feedback, indicated feasibility in cardiovascular practice. Furthermore, ePRO monitoring enhanced patient-physician communication and clarity of physicians’ explanations of treatment as well as increased patient knowledge regarding their cardiac conditions. A user-friendly ePRO monitoring system can reduce patient uncertainty regarding disease management and promote patient-centered cardiovascular care.

These findings contrast with those of a recent randomized clinical trial that evaluated the effect of routine PRO assessment in HF care. In the PRO-HF trial, Sandhu et al^21^ reported that routine collection of KCCQ-12 feedback via electronic health records in an HF clinic did not affect patient-reported quality of life or outcomes of care. Furthermore, therapeutic alliance and quality of communication were not enhanced overall by collecting the KCCQ-12 assessments in a substudy of the PRO-HF trial, while the improvement of patient experience regarding the “clinicians’ understanding of patient symptoms” suggested the potential of PRO assessment.^34^ The difference in PRO impact may stem from varying assessment approaches. First, the frequency of PRO assessments in the current study was higher, with monthly ePRO surveys compared with a mean of 2.4 visits per year in the PRO-HF trial.^21^ The disease-specific questionnaires used in the current study, including the KCCQ-12, focused on health status within a timeframe of 2 to 4 weeks.^23,24,25^ Thus, we aimed to capture the nuances and temporal changes in PROs within weeks rather than months and to proactively detect unintended consequences from the intervention. Given that the Japanese health care system provides free hospital access,^35^ monthly ePRO assessments are feasible in outpatient settings in Japan. This approach may not generalize globally. However, remote monitoring, as conducted in oncological fields, could make monthly assessments practical in clinical settings.^17,18,19^ Second, in this study, all ePRO reports were jointly reviewed by patients and physicians to enhance the clinical relevance and application of the collected data. In contrast, it was unclear whether the completed KCCQ-12 assessments were reviewed by the treating clinician in the PRO-HF trial.^21^ This finding suggests that the potential of PRO can be maximized in cardiovascular care, not only by collecting patient-reported data but also by reviewing it at appropriate intervals.

With the worldwide increase in the prevalence of cardiovascular diseases associated with an aging population, previous studies have highlighted the essential nature of patient-centered cardiovascular care for older patients with complex comorbidities.^2,36^ The effective integration of PROs for patients and health care practitioners is warranted to clarify the multifaceted cardiovascular care goals for the older population. A strength of the full study is the codevelopment of a user-friendly electronic survey system and a unique ePRO implementation method, which involves a joint review by patients and physicians through elaborate multidisciplinary discussions. The feasibility and efficacy of the ePRO monitoring system were shown, with all ePRO surveys successfully completed by patients with a median age of 71 years and a relatively high prevalence of comorbidities. Furthermore, patient feedback collected in phase 1 suggested that our method of ePRO assessment could identify patients’ experiences in which they did not fully communicate with their physicians (eTable 4 in Supplement 2) and enhance their understanding of their condition.

PROs are generally expected to provide patient perspectives and promote shared decision-making through an individualized and bidirectional exchange between patients and health care teams.^37^ However, if the PRO is subjectively interpreted solely by the clinician and interactive discussions about the patient’s health status are lacking, relevant health-related issues may be overlooked, resulting in unidirectional care that is based on the clinician’s perspective. The present study indicated that a joint review of PROs using a user-friendly electronic platform can facilitate bidirectional clinical care between older patients and health care practitioners. We believe that the current ePRO monitoring system is an effective PRO implementation strategy with the potential to support patient-centered care in contemporary cardiovascular practice.

### Limitations

This trial has certain limitations. First, the ePRO monitoring system was based explicitly on the Japanese health care environment and is not available in other languages. Therefore, whether the results are generalizable to diverse populations in different health care systems and languages is unclear. Additionally, while monthly ePRO assessments may be applicable to clinical practice in Japan, more data are needed to demonstrate their feasibility in routine cardiovascular care. Second, the ePRO group included patients who completed the KCCQ-12, AFEQT, or SAQ; however, the impact of PRO monitoring on the quality of cardiovascular care may differ across disease-specific questionnaires. The effect of SAQ monitoring could not be sufficiently examined due to the small number of patients with angina. Further investigation of the disease-specific effects of PRO monitoring is necessary to support the use of PROs for optimal care delivery. Third, the evaluation of disease knowledge has limitations, as disease trajectories can vary widely among patients and are often difficult to predict in HF. Other validated and user-friendly assessment tools should be considered. Fourth, a power calculation was not conducted because of the small scale of the pilot trial. Although there was a possibility of being underpowered to detect differences in the changes in the main outcomes between the 2 treatment groups, the ePRO monitoring system significantly improved the PSQ score regarding communication and QOI score regarding treatment, suggesting the observed effects are likely meaningful. Fifth, this trial was designed as open label with a nonblinded outcome assessment. However, the outcomes were assessed based on patient-reported data. Sixth, the effects of the ePRO monitoring system on patient health status or hard end points in clinical cardiovascular practice have not yet been examined. Our limited data on changes in PRO scores in the ePRO group found that PRO scores improved only in the AFEQT subgroup (eTable 8 in Supplement 2). A larger randomized clinical trial is required to assess the effect of ePRO monitoring system implementation on clinical outcomes in cardiovascular practice.

## Conclusions

This multicenter pilot randomized clinical trial demonstrated the feasibility and efficacy of the electronic monitoring of PROs in cardiovascular care. Implementing the ePRO system significantly improved patient-physician communication and the clarity of physicians’ explanations about treatment. These findings suggest that the ePRO monitoring system can support patient-centered care in cardiovascular practice.

## Notes

Supplement 1.
Trial Protocol and Statistical Analysis Plan

Supplement 2. **eTable 1.** Baseline Characteristics of Patients in Phase 1 **eTable 2.** Questionnaire to Assess Patient Satisfaction and Quality of Information Provided by Physician **eTable 3.** Questionnaires to Assess Patient Knowledge About Heart Failure and Atrial Fibrillation **eTable 4.** Qualitative Assessment for the Prototype ePRO System From Patients **eTable 5.** Qualitative Assessment for the Prototype ePRO System From Physicians **eTable 6.** Changes in Patient Satisfaction and Quality of Information Provided by Physician **eTable 7.** Changes in Individual Components of the 5-Items of Patient Satisfaction Questionnaire **eTable 8.** PRO Scores at Baseline and Last Follow-Up in the ePRO Group **eFigure 1.** Evolution of the Electronic Patient-Reported Outcome Survey System **eFigure 2.** Workflow of Electronic Patient-Reported Outcome Monitoring **eFigure 3.** Four Figures Representing Trajectories of Various Illnesses **eFigure 4.** Patients’ Assessment of the Prototype ePRO System Regarding Visualization and Survey Method **eFigure 5.** Example of KCCQ Report Displayed on a Screen **eFigure 6.** Example of AFEQT Report Displayed on a Screen **eFigure 7.** Example of SAQ Report Displayed on a Screen

Supplement 3.
Data Sharing Statement

## References

[1] Walsh MN, Bove AA, Cross RR, ; American College of Cardiology Foundation. ACCF 2012 health policy statement on patient-centered care in cardiovascular medicine: a report of the American College of Cardiology Foundation Clinical Quality Committee. J Am Coll Cardiol. 2012;59(23):2125-2143. doi:10.1016/j.jacc.2012.03.016 22591882

[2] Kim DH, Rich MW. Patient-centred care of older adults with cardiovascular disease and multiple chronic conditions. Can J Cardiol. 2016;32(9):1097-1107. doi:10.1016/j.cjca.2016.04.003 27378591 PMC5003648

[3] Rumsfeld JS, Alexander KP, Goff DC Jr, ; American Heart Association Council on Quality of Care and Outcomes Research, Council on Cardiovascular and Stroke Nursing, Council on Epidemiology and Prevention, Council on Peripheral Vascular Disease, and Stroke Council. Cardiovascular health: the importance of measuring patient-reported health status: a scientific statement from the American Heart Association. Circulation. 2013;127(22):2233-2249. doi:10.1161/CIR.0b013e3182949a2e 23648778

[4] Katsumata Y, Kimura T, Kohsaka S, . Discrepancy in recognition of symptom burden among patients with atrial fibrillation. Am Heart J. 2020;226:240-249. doi:10.1016/j.ahj.2020.03.024 32517853

[5] Arnold SV, Grodzinsky A, Gosch KL, . Predictors of physician under-recognition of angina in outpatients with stable coronary artery disease. Circ Cardiovasc Qual Outcomes. 2016;9(5):554-559. doi:10.1161/CIRCOUTCOMES.116.002781 27531922 PMC5031528

[6] Tran AT, Chan PS, Jones PG, Spertus JA. Comparison of patient self-reported health status with clinician-assigned New York Heart Association classification. JAMA Netw Open. 2020;3(8):e2014319. doi:10.1001/jamanetworkopen.2020.14319 32857144 PMC7455849

[7] Ikemura N, Kohsaka S, Kimura T, . Physician estimates and patient-reported health status in atrial fibrillation. JAMA Netw Open. 2024;7(2):e2356693. doi:10.1001/jamanetworkopen.2023.56693 38393730 PMC10891467

[8] Katsumata Y, Kohsaka S, Ikemura N, . Symptom under-recognition of atrial fibrillation patients in consideration for catheter ablation. JACC Clin Electrophysiol. 2021;7(5):565-574. doi:10.1016/j.jacep.2020.10.016 33358669

[9] Qintar M, Spertus JA, Gosch KL, . Effect of angina under-recognition on treatment in outpatients with stable ischaemic heart disease. Eur Heart J Qual Care Clin Outcomes. 2016;2(3):208-214. doi:10.1093/ehjqcco/qcw016 28239488 PMC5322471

[10] Heidenreich PA, Spertus JA, Jones PG, ; Cardiovascular Outcomes Research Consortium. Health status identifies heart failure outpatients at risk for hospitalization or death. J Am Coll Cardiol. 2006;47(4):752-756. doi:10.1016/j.jacc.2005.11.021 16487840

[11] Ikemura N, Spertus JA, Nguyen DD, . Baseline health status and its association with subsequent cardiovascular events in patients with atrial fibrillation. JACC Clin Electrophysiol. 2023;9(9):1934-1944. doi:10.1016/j.jacep.2023.05.037 37498250

[12] Spertus JA, Jones P, McDonell M, Fan V, Fihn SD. Health status predicts long-term outcome in outpatients with coronary disease. Circulation. 2002;106(1):43-49. doi:10.1161/01.CIR.0000020688.24874.90 12093768

[13] Hindricks G, Potpara T, Dagres N, ; ESC Scientific Document Group. 2020 ESC Guidelines for the diagnosis and management of atrial fibrillation developed in collaboration with the European Association for Cardio-Thoracic Surgery (EACTS): the task force for the diagnosis and management of atrial fibrillation of the European Society of Cardiology (ESC) developed with the special contribution of the European Heart Rhythm Association (EHRA) of the ESC. Eur Heart J. 2021;42(5):373-498. doi:10.1093/eurheartj/ehaa612 32860505

[14] Heidenreich PA, Bozkurt B, Aguilar D, . 2022 AHA/ACC/HFSA Guideline for the management of heart failure: a report of the American College of Cardiology/American Heart Association joint committee on clinical practice guidelines. Circulation. 2022;145(18):e895-e1032. doi:10.1161/CIR.0000000000001063 35363499

[15] Byrne RA, Rossello X, Coughlan JJ, ; ESC Scientific Document Group. 2023 ESC Guidelines for the management of acute coronary syndromes. Eur Heart J. 2023;44(38):3720-3826. doi:10.1093/eurheartj/ehad191 37622654

[16] Yu JY, Goldberg T, Lao N, Feldman BM, Goh YI. Electronic forms for patient reported outcome measures (PROMs) are an effective, time-efficient, and cost-minimizing alternative to paper forms. Pediatr Rheumatol Online J. 2021;19(1):67. doi:10.1186/s12969-021-00551-z 33941208 PMC8091685

[17] Basch E, Deal AM, Kris MG, . Symptom monitoring with patient-reported outcomes during routine cancer treatment: a randomized controlled trial. J Clin Oncol. 2016;34(6):557-565. doi:10.1200/JCO.2015.63.0830 26644527 PMC4872028

[18] Basch E, Schrag D, Henson S, . Effect of electronic symptom monitoring on patient-reported outcomes among patients with metastatic cancer: a randomized clinical trial. JAMA. 2022;327(24):2413-2422. doi:10.1001/jama.2022.9265 35661856 PMC9168923

[19] Basch E, Deal AM, Dueck AC, . Overall survival results of a trial assessing patient-reported outcomes for symptom monitoring during routine cancer treatment. JAMA. 2017;318(2):197-198. doi:10.1001/jama.2017.7156 28586821 PMC5817466

[20] Brown-Johnson C, Calma J, Amano A, . Evaluating the implementation of patient-reported outcomes in heart failure clinic: a qualitative assessment. Circ Cardiovasc Qual Outcomes. 2023;16(5):e009677. doi:10.1161/CIRCOUTCOMES.122.009677 37114990 PMC10192029

[21] Sandhu AT, Calma J, Skye M, . Clinical impact of routine assessment of patient-reported health status in heart failure clinic: the PRO-HF trial. Circulation. 2024;149(22):1717-1728. doi:10.1161/CIRCULATIONAHA.124.069624 38583147 PMC11774328

[22] World Medical Association. World Medical Association Declaration of Helsinki: ethical principles for medical research involving human subjects. JAMA. 2013;310(20):2191-2194. doi:10.1001/jama.2013.281053 24141714

[23] Spertus JA, Winder JA, Dewhurst TA, . Development and evaluation of the Seattle Angina Questionnaire: a new functional status measure for coronary artery disease. J Am Coll Cardiol. 1995;25(2):333-341. doi:10.1016/0735-1097(94)00397-9 7829785

[24] Green CP, Porter CB, Bresnahan DR, Spertus JA. Development and evaluation of the Kansas City Cardiomyopathy Questionnaire: a new health status measure for heart failure. J Am Coll Cardiol. 2000;35(5):1245-1255. doi:10.1016/S0735-1097(00)00531-3 10758967

[25] Spertus J, Dorian P, Bubien R, . Development and validation of the Atrial Fibrillation Effect on QualiTy-of-Life (AFEQT) Questionnaire in patients with atrial fibrillation. Circ Arrhythm Electrophysiol. 2011;4(1):15-25. doi:10.1161/CIRCEP.110.958033 21160035

[26] Kosiborod M, Soto GE, Jones PG, . Identifying heart failure patients at high risk for near-term cardiovascular events with serial health status assessments. Circulation. 2007;115(15):1975-1981. doi:10.1161/CIRCULATIONAHA.106.670901 17420346

[27] Thayaparan AJ, Mahdi E. The Patient Satisfaction Questionnaire Short Form (PSQ-18) as an adaptable, reliable, and validated tool for use in various settings. Med Educ Online. 2013;18:21747. doi:10.3402/meo.v18i0.21747 23883565 PMC3722414

[28] El-Jawahri A, Traeger L, Kuzmuk K, . Prognostic understanding, quality of life and mood in patients undergoing hematopoietic stem cell transplantation. Bone Marrow Transplant. 2015;50(8):1119-1124. doi:10.1038/bmt.2015.113 25961772 PMC4526323

[29] Kitakata H, Kohno T, Kohsaka S, . Prognostic understanding and preference for the communication process with physicians in hospitalized heart failure patients. J Card Fail. 2021;27(3):318-326. doi:10.1016/j.cardfail.2020.10.009 33171293

[30] Murray SA, Kendall M, Mitchell G, Moine S, Amblàs-Novellas J, Boyd K. Palliative care from diagnosis to death. BMJ. 2017;356:j878. doi:10.1136/bmj.j878 28242747

[31] Allen LA, Yager JE, Funk MJ, . Discordance between patient-predicted and model-predicted life expectancy among ambulatory patients with heart failure. JAMA. 2008;299(21):2533-2542. doi:10.1001/jama.299.21.2533 18523222 PMC3623529

[32] Hendriks JM, Crijns HJ, Tieleman RG, Vrijhoef HJ. The atrial fibrillation knowledge scale: development, validation and results. Int J Cardiol. 2013;168(2):1422-1428. doi:10.1016/j.ijcard.2012.12.047 23305860

[33] Desteghe L, Engelhard L, Raymaekers Z, . Knowledge gaps in patients with atrial fibrillation revealed by a new validated knowledge questionnaire. Int J Cardiol. 2016;223:906-914. doi:10.1016/j.ijcard.2016.08.303 27589038

[34] Sandhu AT, Zheng J, Kalwani NM, . Impact of patient-reported outcome measurement in heart failure clinic on clinician health status assessment and patient experience: a substudy of the PRO-HF trial. Circ Heart Fail. 2023;16(2):e010280. doi:10.1161/CIRCHEARTFAILURE.122.010280 36334312 PMC10108581

[35] For understanding of medical service under health insurance: medical department. Article in Japanese. Ministry of Health, Labour and Welfare. Accessed October 10, 2024. https://www.mhlw.go.jp/content/001274394.pdf

[36] Roth GA, Mensah GA, Johnson CO, ; GBD-NHLBI-JACC Global Burden of Cardiovascular Diseases Writing Group. Global burden of cardiovascular diseases and risk factors, 1990-2019: update from the GBD 2019 study. J Am Coll Cardiol. 2020;76(25):2982-3021. doi:10.1016/j.jacc.2020.11.010 33309175 PMC7755038

[37] Goldfarb MJ, Saylor MA, Bozkurt B, ; American Heart Association Council on Clinical Cardiology; Council on Cardiovascular and Stroke Nursing; Council on Hypertension; Council on Lifestyle and Cardiometabolic Health; Council on Peripheral Vascular Disease; and Council on Quality of Care and Outcomes Research. Patient-centered adult cardiovascular care: a scientific statement from the American Heart Association. Circulation. 2024;149(20):e1176-e1188. doi:10.1161/CIR.0000000000001233 38602110

