# Supplementary material for: Electronic Patient-Reported Outcome System Implementation in Outpatient Cardiovascular Care: A Randomized Clinical Trial
Source: JAMA Netw Open. 2025 Jan 14;8(1):e2454084. doi: 10.1001/jamanetworkopen.2024.54084 (PMC11733702; doi:10.1001/jamanetworkopen.2024.54084)
Supplement: Supplement 1. — Trial Protocol and Statistical Analysis Plan [file jamanetwopen-e2454084-s001.pdf]

Final protocol

Clinical Research Protocol

Study of usefulness of ePRO in cardiovascular disease

Ethics Review Committee Approval Number: 20221093

UMIN ID: UMIN000049251

Version	Date Created	Prepared By	Reason	Distribution
Version 1.0	April 28, 2022	Yoshinori Katsumata	New document	Keio University School of Medicine
Version 1.1	August 31, 2022	Yoshinori Katsumata	Post IRB pre-review	Keio University School of Medicine
Version 1.2	September 5, 2022	Yoshinori Katsumata	Post IRB pre-review	Keio University School of Medicine
Version 1.3	October 6, 2022	Yoshinori Katsumata	Post IRB pre-review	Keio University School of Medicine

Table of Contents	
1. Overview	Page 4
2. Background of Study	Page 5
3. References	Page 7
4. Study Design	Page 8
5. Criteria for Inclusion, Exclusion, and Discontinuation	Page 11
6. Study Treatment	Page 12
7. Efficacy Evaluation	Page 12
8. Safety Evaluation	Page 13
9. Statistical Analysis	Page 13
10. Direct Access to Source Data and Documents	Page 14
11. Monitoring and Auditing	Page 15
12. Ethics	Page 15
13. Handling and Record Preservation of Data and Samples	Page 17
14. Economic Burden and Other Measures for Participants	Page 17
15. Arrangements for Publishing Research Results	Page 17
16. Addendum	Page 17

1. Overview

1.1 Study Information

Project Title: Study of usefulness of ePRO in cardiovascular disease
Ethics Review Committee Approval Number: 20221093
UMIN ID: UMIN000049251

1.2 Principal Investigator

Principal Investigator: Masahiro Jinzaki
Affiliation: Keio University School of Medicine
Department: Diagnostic Radiology
Position: Professor
Address: 35 Shinanomachi, Shinjuku-ku, Tokyo, Japan
Phone Number: 03-5269-9054 (Weekdays 8:40 - 17:00)
Email: jinzaki@rad.med.keio.ac.jp

1.3 Study Office

Contact Person: Yoshinori Katsumata
Affiliation: Keio University School of Medicine
Department: Sports Medicine Comprehensive Center
Position: Senior Lecturer
Location: 35 Shinanomachi, Shinjuku-ku, Tokyo, Japan
Phone Number: 03-5269-9054 (Weekdays 8:40 - 17:00)
Email: goodcentury21@keio.jp

1.4 Contracted Facilities

None

1.5 Implementation Facilities and Principal Investigator

Keio University School of Medicine, Yoshinori Katsumata
University of Occupational and Environmental Health, Masaharu Kataoka
National Hospital Organization Saitama Hospital, Tomohiko Ono
Hino municipal Citizen’s Hospital, Satoshi Shoji

2. Background of Study

2.1 Background, study objective and meaning

2.1.1 Background

The number of cardiovascular patients is rapidly increasing as the population ages. In particular, the number of patients with heart failure is on the rise worldwide (1).

According to the epidemiological survey by Japanease Cardiology Society, the number of heart failure hospitalization has been increased by 10,000 per year and the prevalence of heart failure is about 1000,000 persons today. The number of HF patients in Japan will continue to increase (2).

In the patients with cardiovascular disease such as HF, atrial fibrillation (AF) and coronary artery disease (CAD), behavioral pattern in life, daily activity, quality of sleep and mental state have a significant impact on their disease condition (3-5).

For modifying their lifestyle and preventing disease progression, it is vital to assess Patient-Reported Outcome (PRO) that can quantify symptom, physical function, anxiety, satisfaction and so on.

The principal aim in the management of cardiovascular disease in recent years has extended beyond merely reducing cardiovascular events; it has also encompassed enhancing the quality of life for individual patients. PRO measures (e.g. Kansas City Cardiomyopathy Questionnaire (KCCQ), Atrial Fibrillation Effect on QualiTy-of-life (AFEQT) and Seattle Angina Questionnaire (SAQ)) and Short Physical Performance Battery have been regarded as important endpoints in clinical research (6).

We previously established multicenter registries for patients with cardiovascular disease, collecting PROs using disease-specific questionnaires (7) and reported the importance of PRO assessment in clinical settings. From this registry, we found that discrepancies in symptom recognition between patients and physicians often existed in daily practice (8). Furthermore, physicians' under-recognition of patients' symptoms was associated with less aggressive treatment, such as catheter ablation (9,10).

It is imperative to develop the electronic PRO (ePRO) survey system utilizing IoT for implementing PRO assessment in clinical practice.

As ePRO becomes more widespread, it is expected that medical care, which was often viewed as a service provided unilaterally by doctors, will be transformed into a service that enables mutual communication between doctors and patients.

2.1.2 Study Objective

To investigate whether visualizing patient health status with ePRO monitoring for cardiovascular patients improve patient satisfaction.

2.1.3 Meaning

Discrepancies in recognition between patients and physicians in healthcare have been reported both domestically and internationally to affect the quality of care and treatment outcomes. Therefore, it is important to understand this discordant recognition between patients and physicians, and as a foundation, it is necessary to understand the patient perspective in clinical practice. Traditionally, healthcare has often been perceived as a service provided unidirectionally from the physician's side, but with the spread of ePRO, the patient perspective is becoming visible, transforming healthcare into a service that enables bidirectional communication between patients and physicians. Additionally, by reducing the omission of patients' subjective symptoms, earlier and more appropriate therapeutic interventions can be expected. It is also characteristic of ePRO that it includes items such as anxiety about treatment and satisfaction, and understanding these aspects may lead to a more holistic clinical practice that comprehensively understands and values the patient's values and quality of life (QoL).

2.2 Previous evidence and validity of the Research Implementation

Concordant understanding of disease condition and treatment choice is necessary for the improvement of the quality of care and treatment success. We have previously reported that the discordance in recognition of symptoms between patients and physicians often existed in daily practice. One of the supportive methods for shared understanding of patient health status between patients and physicians is the implementation of ePRO, which can make patient information visible to physicians.

The routine collection of ePRO is recommended with a Class I indication in the European Society of Cardiology guidelines. We have previously introduced PROs into disease registries for heart failure, atrial fibrillation, and angina pectoris, emphasizing the importance of making treatment decisions based on patient-reported health status. There is a discrepancy between the symptoms perceived by patients and those recognized by physicians, resulting in the insufficient provision of advanced but invasive medical care (8,9). A previous study in the field of oncology have also reported that the implementation of PRO assessment improved outcomes (1), and verification of this in the field of cardiology remains a critical issue.

For the implementation of PROs, which quantify (numerically measure) patient-reported symptoms in medical care, the development of digitized ePROs utilizing IoT is essential. We have advanced the development of digital versions of the AFEQT for atrial fibrillation, the

KCCQ for heart failure, and the SAQ for stable angina, which have been previously employed in the cardiovascular disease domain. Using these prototypes, we conducted a pilot study with 10 patients. As a result, we received positive feedback from patients, such as being able to convey information they couldn't fully express during consultations and having a clearer understanding of whether their clinical course was proceeding successfully.

2.3 Participant Risk and Benefit

Risk: There is no financial burden. The time required for ePRO and questionnaires is approximately 5 minutes. In the event of adverse events, appropriate medical care will be provided. Participant privacy is respected through linked anonymization. The participant list is password-protected by the information manager, stored on a hard disk, and kept in the Sports Medicine Center office. Specifically, personal information such as the participant's name and medical record number, as well as ePRO and questionnaire results obtained using wearable devices, will be accessed and viewed through the dedicated website for this study. This allows for conversations between physicians and participants regarding trends during consultations, contributing to the improvement of patient satisfaction verified in this study. Researchers can then download and anonymize the data for storage and use within the office's hard disk as anonymized research data.

Only authorized research personnel and maintenance developers of this study system with access rights to website for this trial can register and have access to the personal data of participants. There is no risk of privacy infringement because all downloaded research data undergo anonymization processing.

Benefits: The results of ePRO are calculated immediately and visualized on the device, allowing the current scores to be shared between physicians and subjects during consultations. There is no compensation for participation in this study.

2.4 Compliance with Regulations and Guidelines

The research will be conducted in accordance with the Helsinki Declaration and ethical guidelines for biomedical and medical research involving human subjects.

2.5 Target Patient

Refer to Section 5 for criteria for selection, exclusion, and discontinuation of subjects

3. References

1. Parizo JT, Kohsaka S, Sandhu AT, et al; Trends in Readmission and Mortality Rates Following Heart Failure Hospitalization in the Veterans Affairs Health Care System From 2007 to 2017. JAMA Cardiol. 2020;5(9):1042-1047.
2. Yasuda S, Miyamoto Y, Ogawa H. Current Status of Cardiovascular Medicine in the Aging Society of Japan. Circulation. 2018;138(10):965-967.
3. Shiraishi Y, Kohsaka S, Abe T, et al; Tokyo CCU Network Scientific Committee. Impact of Triggering Events on Outcomes of Acute Heart Failure. Am J Med. 2018;131(2):156-164.e2.
4. Li X, Xue Q, Wang M, et al; Adherence to a Healthy Sleep Pattern and Incident Heart Failure: A Prospective Study of 408 802 UK Biobank Participants. Circulation. 2021;143(1):97-99.
5. Matsuda R, Kohno T, Kohsaka S, et al; Psychological disturbances and their association with sleep disturbances in patients admitted for cardiovascular diseases. PLoS One. 2021;16(1):e0244484.
6. Kitzman DW, Whellan DJ, Duncan P, et al; Physical Rehabilitation for Older Patients Hospitalized for Heart Failure. N Engl J Med. 2021;385(3):203-216.
7. Ikemura N, Spertus JA, Kimura T, et al; Cohort profile: patient characteristics and quality-of-life measurements for newly-referred patients with atrial fibrillation-Keio interhospital Cardiovascular Studies-atrial fibrillation (KiCS-AF). *BMJ Open*. 2019;9(12):e032746.
8. Katsumata Y, Kimura T, Kohsaka S, et al; Discrepancy in recognition of symptom burden among patients with atrial fibrillation. *Am Heart J*. 2020;226:240-249.
9. Ikemura N, Kohsaka S, Kimura T, et al; Physician Estimates and Patient-Reported Health Status in Atrial Fibrillation. *JAMA Netw Open*. 2024;7(2):e2356693.
10. Katsumata Y, Kohsaka S, Ikemura N, et al; Symptom Under-Recognition of Atrial Fibrillation Patients in Consideration for Catheter Ablation: A Report From the KiCS-AF Registry. *JACC Clin Electrophysiol*. 2021;7(5):565-574.

4. Study Design

4.1 Outcome

(1) Primary Outcome

Patient satisfaction calculated by Likert-Scale

(2) Secondary outcome

Assessment of well-being, patient preferences for shared decision-making using questionnaires. Qualitative assessment of ePRO monitoring in ePRO group.

(3) exploratory outcome

Factors associated with improved patient satisfaction in ePRO group

4.2 Study method

In this study, participants will be randomly assigned to ePRO monitoring or usual care. We will explain this research face-to-face to the patients and obtain electronic consent. Prior to randomization, patients with HF, AF or CAD will be assigned to KCCQ-12, AFEQT or SAQ respectively. For patients with two or more of these three coexisting cardiovascular diseases, treating physicians will assign an appropriate PRO measure based on their primary cardiovascular disease.

Participants will be randomly assigned to ePRO group or control group. After randomization, they are instructed to respond to the questionnaires for outcome assessment. Patients assigned to ePRO group are also asked to respond to physician assigned PRO measures.

All patients will be followed up on an outpatient basis every 4 weeks (± 2 weeks) for a total of 5 visits. At every visit, patients in the ePRO group will receive a smart tablet and answer for the assigned questionnaire (KCCQ-12, AFEQT or SAQ) before entering the examination room. The reports of PROs will be displayed on smart tablets and shared between physicians and patients during medical examination. Patients in the control group received standard care in accordance with the latest clinical guidelines. All patients will be asked to respond to the questionnaires for outcome assessment at the fifth visit

In addition, the following information will be collected from participants through interviews or electronic medical records:

(1) Patient information: the date of birth, age, sex, height, weight, Vital signs (systolic blood pressure, diastolic blood pressure, heart rate, respiratory rate), family history, past history of illness, medication history.
(2) Blood tests: White blood cell count (WBC), red blood cell count (RBC), hemoglobin concentration (Hb), hematocrit value (Ht), mean corpuscular volume (MCV), mean corpuscular hemoglobin (MCH), mean corpuscular hemoglobin concentration (MCHC), platelet count (PLT),

Total protein (TP), creatinine (CRE), uric acid (UA), sodium (Na), potassium (K), chloride (Cl), calcium (Ca), aspartate aminotransferase (AST), alanine aminotransferase (ALT), gamma-glutamyltransferase (γ-GT), alkaline phosphatase (ALP), lactate dehydrogenase (LDH), total cholesterol (Tcho), triglycerides (TG), LDL cholesterol (LDL-cho), HDL cholesterol (HDL-cho), non-HDL cholesterol (calculated value), total bilirubin (T-BIL), glucose (GLU), HbA1c, brain natriuretic peptide (BNP), NT-proBNP, Prothrombin time-international normalized ratio (PT-INR), Activated partial thromboplastin time (APTT), D-dimer.

(3) Electrocardiogram
(4) Echocardiography
(5) Endoscope test
(6) Imaging tests (computed tomography, magnetic resonance imaging, radioisotope imaging, chest X-ray (presence of pleural effusion, CTR)
(7) Exercise test

4.3 Data

4.3.1 Original data

Data will be collected from electronic PRO survey, questionnaire for outcome assessment and electronic medical records.

4.3.2 Data Collection

Data will be collected using electronic devices (e.g. smart tablets) for PRO and questionnaires. These data will be collected five times during consultations (every 4 weeks ±2 weeks). Patient background information will be obtained directly from the subjects, and medical information will be gathered from electronic medical records.

4.3.3 Data Management and Record Keeping

The data obtained in this study will not be used for other research purposes without the consent of the patients. Strict anonymization of the collected data will be enforced, and the data will be stored on password-protected personal computers in the Department of Cardiology and the Sports Medicine Center (secured with locks).

While the possibility of personal information leakage is very low, it cannot be entirely ruled out. To protect personal information, the data analyzed will be in a de-identified form, with proper

management of the correspondence table. Additionally, meticulous care will be taken in data management to prevent any data leakage.

4.4 Bias

Adaptive randomization method will be applied to reduce intervention bias. Due to the nature of the intervention, blinding is not feasible, and thus the trial will be conducted with an open-label design.

4.5 Study Participants Compliance

Not applicable.

4.6 Enrollment and Registration of Study Participants

(1) Confirm that candidate patients meet all inclusion criteria and do not meet any exclusion criteria.
(2) Provide an explanation of the study and obtain consent electronically.
(3) Assign an anonymized ID to subjects who have given consent. Randomly determine whether the subject will respond to ePRO or not on a computer before starting the study.

4.7 Study Period

From the date of study approval to March 31, 2024.

4.8 Study Interruption or Termination

The investigator shall consider whether to continue the study based on the study protocol in the following cases:

(1) If changes to the study protocol are necessary, and the implementing medical institution cannot accommodate these changes.
(2) If the head of the implementing medical institution issues a directive to modify the study based on the opinion of the ethics committee, and the investigator does not agree to this directive.
(3) If the ethics committee states that the study should not continue, and the head of the implementing medical institution orders the study to be terminated.
(4) When the principal investigator changes due to transfer or other reasons, and the study is

paused until a new principal investigator can take over the study.

5. Criteria for Inclusion, Exclusion, and Discontinuation

5.1 Inclusion Criteria

Adult cardiovascular disease patients aged 18 and older who are currently receiving outpatient treatment. For angina and myocardial infarction, patients who have previously undergone catheter examinations; for heart failure, patients who meet the Framingham criteria and have a BNP level exceeding 100 pg/ml at the time of case registration; for atrial fibrillation, patients diagnosed with atrial fibrillation on a 12-lead electrocardiogram.

5.2 Exclusion Criteria

Individuals deemed unsuitable as subjects for this study by the principal or co-investigators.
Individuals who are unable to operate electronic devices such as tablets.

5.3 Discontinuation Criteria

The principal investigator or sub-investigators will discontinue the study for a subject if any of the following conditions are identified after subject registration:

(1) An adverse event occurs, and the principal investigator or sub-investigators determine that the study should be discontinued.
(2) The subject requests to withdraw from the study.
(3) It is determined that the subject is inappropriate for inclusion in the study.
(4) It becomes evident that the necessary observations and tests cannot be conducted due to the subject’s circumstances.
(5) Any other reason where the principal investigator or sub-investigators judge that the collection of questionnaire data should be discontinued.

Reasons for Settings:

(1) was established to ensure safety considerations.
(2) and (3) was set as part of the informed consent process, acknowledging the subjects' rights.
(4) was established because subjects who cannot undergo necessary observations should be withdrawn from the study promptly.
(5) was set in consideration of any other situations requiring the discretion of the principal

investigator or sub-investigators to discontinue the study.

6. Study Treatment

6.1 Contents of the Study Treatment

The system used to collect ePRO questionnaires in this clinical trial is as follows:

Name: Smart-Pro (tentative name)

System Provider: Chubu Electric Power Co., Inc.

Usage Purposes:

Collection, accumulation, and utilization of ePRO (AFEQT, KCCQ, SAQ) data

Tool for obtaining electronic informed consent.

Random allocation tool for assigning patients to either the ePRO group or the control group (ePRO non-response group).

6.2 Concomitant Therapy

Standard treatment for atrial fibrillation, heart failure, and angina pectoris will be provided.

6.3 Compliance

Not applicable

7. Efficacy Evaluation

7.1 Efficacy Evaluation Indicators

7.2 Methods for Evaluating, Recording, and Analyzing Efficacy Evaluation Indicators

ePRO and questionnaire data will be collected using electronic devices and stored in the Smart-Pro (tentative name) system on servers contracted by Chubu Electric Power Co., Inc. The data will be displayed on a dedicated web interface to improve patient satisfaction through result viewing during consultations between physicians and patients. Researchers will extract data from the server and manage it as research data. The data will be matched with patient background and medical data, and changes in the utilization of ePRO will be analyzed. Planned analyses include paired t-tests and logistic regression analyses.

8. Safety Evaluation

8.1 Adverse Events (AE)

The time required to answer the questionnaire is approximately 5 minutes each time, which may cause mental distress or time constraints.

8.2 Serious Adverse Events (SAE)

Not anticipated.

8.3 Procedures for Collecting, Recording, and Reporting AE/SAE

Not anticipated.

8.4 Follow-up of Patients After the Occurrence of Adverse Events and Health Damage Compensation

Health Damage Compensation: None

In this study, the safety of patients will be ensured by fully considering the contents described in "8.1 Adverse Events." If medical care is needed due to an adverse event, the subject will be informed, and appropriate medical care will be provided. In addition, an emergency response system will be in place to address unforeseen incidents.

9. Statistical Analysis

9.1 Number of Registered Cases

A total of 50 cases will be assigned to either the ePRO response group or the non-response group.

Basis: The number of participants was determined based on the number that can be registered within the set research period by the research group. Therefore, no power analysis was conducted.

9.2 Analysis of Results

Analyses will be performed using multivariate analysis, unpaired t-tests, etc.

9.3 Significance Level

A two-sided significance level of 5% will be used for hypothesis testing. When calculating confidence intervals, a two-sided 95% confidence interval will be applied.

9.4 Trial Termination Criteria

The principal investigator will consider the continuation of the study based on the implementation

plan of the medical institution in the following cases:

If changes to the implementation plan are required, and the medical institution cannot accommodate these changes.
If the head of the medical institution gives instructions for modifications based on the opinion of the ethics committee, and the principal investigator does not accept these modifications.
If the ethics committee states that the study should not continue, and the head of the medical institution instructs the study to be terminated.
If the responsible physician is changed due to reasons such as transfers, and the new responsible physician cannot continue the study until they are able to do so (suspension).

9.5 Handling of Trial Data

To strictly manage and adequately protect the personal information of participants, the data handled in this study will be anonymized using a correspondence table and used only as information that cannot identify specific individuals. During data analysis, personal information such as addresses and names will not be handled. The correspondence table linking participants with their newly assigned anonymized IDs will be strictly managed by the personal information manager of this study. When publishing the research results, personal information will not be disclosed. Chubu Electric Power Company will provide the cloud system for collecting ePRO and survey responses, as well as obtaining electronic consent, solely for research purposes. They will not analyze or use the data beyond the scope agreed upon for this study. Researchers will download the data from the system, store and manage it within the institution for research purposes, and analyze the data accordingly.

9.6 Deviations from the Statistical Analysis Plan

Any results derived from analyses that deviate from the initial statistical analysis plan will be clearly explained.

9.7 Analysis Population

The primary and secondary endpoints in this study will be analyzed using the largest analysis cohort, the full analysis set (FAS), as the main analysis.

10. Direct Access to Source Data and Documents

The source data will be electronic data downloaded for research and analysis purposes. Direct access to the source data and documents will be permitted during review by the ethics review committee.

11. Monitoring and Auditing

None.

12. Ethics

The study will be conducted in accordance with the Declaration of Helsinki and the Ethical Guidelines for Life Sciences and Medical Research Involving Human Subjects.

12.1 Informed Consent

Informed consent will be obtained electronically from the research subjects themselves before conducting the study. Specifically, healthcare professionals involved in this study will conduct face-to-face "identity verification," "confirmation that the selection, exclusion, and discontinuation criteria are met," and "selection of the appropriate questionnaire based on the subject's symptoms (AFEQT for atrial fibrillation, KCCQ for heart failure, SAQ for angina)" within the hospital. They will register the basic information (name, patient ID number, type of questionnaire, etc.) of the potential subjects into the "Smart Pro (tentative name)" system.

Next, the potential subjects will review the explanation document and consent item list of this study on their personal page linked to the basic information registered in the "Smart Pro (tentative name)" system, and after understanding them, they will complete the electronic informed consent procedure by checking the checkboxes for the explanation items and pressing the consent button based on their free will.

After the study begins, the responsible physician and other medical professionals involved in this study will inform the subjects how to access the electronic materials related to the explanation and consent content they received and agreed to. They will also provide information on how to contact the consultation office (established at the study office) for any inquiries related to the study and the consent withdrawal procedure.

If consent withdrawal occurs after study participation, the study office will instruct Chubu Electric Power to modify the data accordingly. This instruction does not include the deletion of operational log data necessary for addressing system failures or incidents, but the data will be rendered unviewable on the system screens. The update result of data deletion will be confirmed by a screenshot report from Chubu Electric Power. Chubu Electric Power will update the data stored in the "Smart Pro (tentative name)" system according to the instructions and receive confirmation of the implementation from the study office. The office established at the study office for inquiries is described in "12.4 Handling

Inquiries from Participants and Their Associates."

12.1.1 Consent by a Representative

Reasons for requiring cooperation through a representative: Not applicable

Selection policy for representatives: Not applicable

Explanation items to representatives: Not applicable

12.1.2 Informed Assent

Not applicable

12.2 Reporting to the Head of the Research Institution

Annual reports and reports of suspension, termination, or completion will be made in accordance with the ethical guidelines.

12.3 Disclosure of Research Information

The clinical trial will be registered with UMIN.

12.4 Handling Inquiries from Participants and Their Associates

Participants can contact the person responsible for the study below to directly discuss any concerns. If they wish to withdraw their consent, they can also directly discuss this with the responsible person below.

Medical Institution: Keio University Hospital

Location: 35 Shinanomachi, Shinjuku-ku, Tokyo

Phone Number: 03-5269-9054, Weekdays 8:40 - 17:00

Department: Sports Medicine Comprehensive Center

Responsible Physician: Yoshiki Katsumata

12.5 Conflict of Interest

The costs required for this study will be covered by the Sports Medicine Comprehensive Center laboratory expenses, JST research funds, and AI Hospital project funds. "Smart Pro (tentative name)" is provided free of charge by Chubu Electric Power Company, and no financial support is received. A joint research agreement will be signed with Chubu Electric Power Company. The conflict of interest management committees of each research institution will determine whether fair research can be

conducted.

12.6 Post-Study Arrangements for Participants

None

12.7 Handling Significant Genetic Findings

Not applicable

13. Handling and Record Preservation of Data and Samples

Data will be stored on personal computers (password-protected) in the Department of Cardiovascular Medicine and Sports Medicine Comprehensive Center (with locking facilities). The data will be kept for at least five years from the date of the research completion report or three years from the final research result report, whichever is later. After that, the obtained data will be stored on a dedicated PC after unlinkable anonymization, confirming that there is no information identifying individuals, with the responsible researcher and the personal information manager. The correspondence table used for anonymization will be completely deleted from the hard disk. If the data can be stored and managed within the locked personal computers in the Department of Cardiovascular Medicine and Sports Medicine Comprehensive Center before the five-year or three-year period mentioned above, the data in the Chubu Electric Power research system can be deleted.

13.1 Possibility of Using Data and Samples in Future Research and Providing Them to Other Institutions

Not applicable

14. Economic Burden and Other Measures for Participants

There is no economic burden. Insurance and other measures are not applicable.

15. Arrangements for Publishing Research Results

During the analysis, data will be separated from personal identifiers, and when publishing research results, personal identification will not occur.

16. Addendum

Not applicable

Usefulness of ePRO in Cardiovascular Disease

Statical Analysis Protocol

Ver. 1.0

Principal Investigator

Keio university school of medicine, Institute for Integrated Sports Medicine

Yoshinori Katsumata

Revision History:

Ver1.0 Created on April 28, 2022

1. Study Objective

To investigate whether visualizing patient health status with electrical Patient-Reported Outcome (ePRO) monitoring for cardiovascular patients improves patient satisfaction.

1.1. Primary endpoint

Patient satisfaction calculated by Likert-Scale

1.2. Secondary endpoint

1. Assessment of well-being, patient preferences for shared decision-making using questionnaires.
2. Qualitative assessment of ePRO monitoring in ePRO group.

1.3. Exploratory endpoint

Factors associated with improved patient satisfaction in ePRO group

1.4. Safety Evaluation

The time required to answer the questionnaire is approximately 5 minutes each time, which may cause mental distress or time constraints.

1.5. Sample size

Target Sample Size: 50 subjects (25 subjects × 2 groups)

Because this was a pilot randomized controlled trial, formal sample size calculations were not performed.

2. General Considerations in Statistical Analysis

2.1. Interim Analysis

No interim analysis will be conducted.

2.2. Data Monitoring Committee

A Data Monitoring Committee will not be established. Decisions regarding data handling will be made through consultation between the Principal Investigator and the Lead Statistician.

2.3. Summary Statistics

For continuous variables, the following summary statistics will be provided: sample size, mean, median, standard deviation, minimum, 25th percentile, 75th percentile, and maximum. For categorical variables, the sample size, frequency, and percentage will be reported.

2.4. Significance Level and Confidence Interval

A two-sided significance level of 5% will be used for hypothesis testing. When calculating confidence intervals, a two-sided 95% confidence interval will be applied.

2.5. Adjustment for Multiplicity

No adjustment for multiplicity will be applied in the analyses of efficacy and safety.

2.6. General Rules for Data Handling

【Rules for Calculating Days and Periods】

- Unless otherwise specified, the number of days will be calculated by subtracting the start date from the end date and adding 1.
- When converting days into years, months, or weeks, 1 year will be considered as 365.25 days, 1 month as 30.4375 days, and 1 week as 7 days.

【Rules for Significant Figures】

- For mean, standard deviation, median, and interquartile range, one decimal place lower than the raw data will be used (i.e., round off the second decimal place).
- Maximum and minimum values will retain the same number of decimal places as the raw data.
- Percentages will be displayed to one decimal place (e.g., 12.3%), rounding off the second decimal place.
- When calculating statistical measures such as mean, standard deviation, or median, rounding will not be applied during intermediate calculations; only the final result will be rounded.
- P-values will be reported to three decimal places (rounding the fourth decimal place). If rounding results in a value of 0.000, it will be displayed as < 0.001.

2.7. Data Handling

In principle, data handling decisions will be made by the Principal Investigator. If questions or uncertainties arise, the final decision will be made in consultation with the Lead Statistician. The handling of missing values and variable transformations is outlined below:

- Missing Data: No special statistical imputation methods will be applied to missing data. An unpaired t-test will be performed on complete data excluding missing data.
- Outlier Data: The handling of outlier data will be determined by the Data Handling Committee (comprising the Principal Investigator and the Lead Statistician).
- Variable Transformation: If variable transformations (e.g., square root or logarithmic transformations) are applied, details will be documented in the "Analysis Results Report."

2.8. Amendments to the Statistical Analysis Plan

Modifications to the study protocol that require changes to this Statistical Analysis Plan will result in a revised plan, with details of the amendments recorded in the "Revision History of the Statistical Analysis Plan."

2.9. Final Analysis

The final analysis will be conducted only after data has been obtained for all cases and both cases and data have been locked. Statistical analysis will not be conducted prior to this point. The Lead Statistician will compile the "Analysis Results Report" and submit it to the Principal Investigator for approval.

3. Study method

In this study, participants will be randomly assigned to ePRO monitoring or usual care. We will explain this research face-to-face to the patients and obtain electronic consent. Prior to randomization, patients with HF, AF or CAD will be assigned to KCCQ-12, AFEQT or SAQ respectively. For patients with two or more of these three coexisting cardiovascular diseases, treating physicians will assign an appropriate PRO measure based on their primary cardiovascular disease. Participants will be randomly assigned to ePRO group or control group. After randomization, they are instructed to respond to the questionnaires for outcome assessment. Patients assigned to ePRO group are also asked to respond to physician assigned PRO measures.

All patients will be followed up on an outpatient basis every 4 weeks (± 2 weeks) for a total of 5 visits. At every visit, patients in the ePRO group will receive a smart tablet and answer for the

assigned questionnaire (KCCQ-12, AFEQT or SAQ) before entering the examination room. The reports of PROs will be displayed on smart tablets and shared between physicians and patients during medical examination. Patients in the control group received standard care in accordance with the latest clinical guidelines. All patients will be asked to respond to the questionnaires for outcome assessment at the fifth visit

4. Analysis Cohort

The primary and secondary endpoints in this study will be analyzed using the largest analysis cohort, the full analysis set (FAS), as the main analysis, applying an intention-to-treat approach. Sensitivity analysis will be conducted using the per-protocol set (PPS), a cohort adhering strictly to the study protocol, if necessary.

4.1. Full Analysis Set (FAS)

The FAS will include all subjects enrolled in this study. However, data from subjects with significant protocol violations (e.g., lack of consent, enrollment outside the contract period, or absence of efficacy data) will be excluded.

4.2. Per Protocol Set (PPS)

The PPS will consist of subjects from the FAS, excluding cases with protocol deviations related to study methods or concomitant therapies, including:

・Violation of inclusion criteria
・Violation of exclusion criteria
・Protocol deviations (e.g., failure to complete two scheduled exercise tests).

4.3. Handling of Subjects

The Principal Investigator will make decisions regarding the handling of enrolled subjects. If any issues arise concerning subject handling, the matter will be resolved through consultation with the Lead Statistician.

5. Plan for Analysis of Subject Breakdown and Overall Exposure Status

5.1. Breakdown of Subjects

The following items will be summarized, showing frequencies and percentages. The results will be presented in figures and tables.

・Enrolled cases
・Cases included in the analysis for the primary endpoint
・Cases included in the analysis for each secondary endpoint
・Cases with violations of inclusion criteria
・Cases with violations of exclusion criteria
・Discontinued cases
・Dropouts
・Completed cases

6. Efficacy Analysis

6.1. Primary endpoint

Patient satisfaction calculated by Likert-Scale. Patient satisfaction, assessed using a Likert scale were compared between the two groups. Mean changes from baseline to the final follow-up were analyzed using unpaired t-tests.

6.2. Secondary endpoint

1. Assessment of well-being, patient preferences for shared decision-making using questionnaires.
2. Qualitative assessment of ePRO monitoring in ePRO group.

The changes in Patient Satisfaction Questionnaire (PSQ) score and Quality of Information (QoI) related to treatment and prognosis were compared between the two groups. Mean changes from baseline to the final follow-up were analyzed using unpaired t-tests.

6.3. Exploratory endpoint

Factors associated with improved patient satisfaction in ePRO group
For qualitative data, the changes from before to after the intervention were compared.

7. Safety analysis

7.1. Adverse Events

The incidence and number of cases of adverse events will be summarized, and the occurrence rate along with its 95% confidence interval will be calculated. The two-sided 95% confidence interval will be determined using an exact method based on the F-distribution.

8. Statistical analysis implementation system and environment

8.1. Chief of Statistical Analysis

Keio University School of Medicine, Department of Cardiology

Shun Kohsaka, MD, PhD

8.2. Director of statistical analysis

Keio University School of Medicine, Institute for Integrated Sports Medicine

Yuki Muramoto

8.3. Dataset/Program

Statistical analysis will be conducted using SAS datasets generated from a finalized database, following the establishment of "Subject Handling Criteria" and after data review. In accordance with the SOP of the Biostatistics Department at the Clinical Research Promotion Center, Keio University Hospital, all data analysis programs will undergo development and validation. The data for primary and secondary endpoints will be locked first, followed by the sequential locking of exploratory endpoint data after final data confirmation.
