# Supplementary material for: Electronic Patient-Reported Outcome System Implementation in Outpatient Cardiovascular Care: A Randomized Clinical Trial
Source: JAMA Netw Open. 2025 Jan 14;8(1):e2454084. doi: 10.1001/jamanetworkopen.2024.54084 (PMC11733702; doi:10.1001/jamanetworkopen.2024.54084)
Supplement: Supplement 2. — eTable 1. Baseline Characteristics of Patients in Phase 1 eTable 2. Questionnaire to Assess Patient Satisfaction and Quality of Information Provided by Physician eTable 3. Questionnaires to Assess Patient Knowledge About Heart Failure and Atrial Fibrillation eTable 4. Qualitative Assessment for the Prototype ePRO System From Patients eTable 5. Qualitative Assessment for the Prototype ePRO System From Physicians eTable 6. Changes in Patient Satisfaction and Quality of Information Provided by Physician eTable 7. Changes in Individual Components of the 5-Items of Patient Satisfaction Questionnaire eTable 8. PRO Scores at Baseline and Last Follow-Up in the ePRO Group eFigure 1. Evolution of the Electronic Patient-Reported Outcome Survey System eFigure 2. Workflow of Electronic Patient-Reported Outcome Monitoring eFigure 3. Four Figures Representing Trajectories of Various Illnesses eFigure 4. Patients’ Assessment of the Prototype ePRO System Regarding Visualization and Survey Method eFigure 5. Example of KCCQ Report Displayed on a Screen eFigure 6. Example of AFEQT Report Displayed on a Screen eFigure 7. Example of SAQ Report Displayed on a Screen [file jamanetwopen-e2454084-s002.pdf]

Supplemental Online Content

Yamashita S, Katsumata Y, Kohsaka S, et al. Electronic patient-reported outcome system implementation in outpatient cardiovascular care: a randomized clinical trial. *JAMA Netw Open*. 2025;8(1):e2454084.
doi:10.1001/jamanetworkopen.2024.54084

eTable 1. Baseline Characteristics of Patients in Phase 1
eTable 2. Questionnaire to Assess Patient Satisfaction and Quality of Information Provided by Physician
eTable 3. Questionnaires to Assess Patient Knowledge About Heart Failure and Atrial Fibrillation
eTable 4. Qualitative Assessment for the Prototype ePRO System From Patients
eTable 5. Qualitative Assessment for the Prototype ePRO System From Physicians
eTable 6. Changes in Patient Satisfaction and Quality of Information Provided by Physician
eTable 7. Changes in Individual Components of the 5-Items of Patient Satisfaction Questionnaire
eTable 8. PRO Scores at Baseline and Last Follow-Up in the ePRO Group
eFigure 1. Evolution of the Electronic Patient-Reported Outcome Survey System
eFigure 2. Workflow of Electronic Patient-Reported Outcome Monitoring
eFigure 3. Four Figures Representing Trajectories of Various Illnesses
eFigure 4. Patients' Assessment of the Prototype ePRO System Regarding Visualization and Survey Method
eFigure 5. Example of KCCQ Report Displayed on a Screen
eFigure 6. Example of AFEQT Report Displayed on a Screen
eFigure 7. Example of SAQ Report Displayed on a Screen

This supplemental material has been provided by the authors to give readers additional information about their work.

© 2025 Yamashita S et al. *JAMA Network Open.*

eTable 1. Baseline characteristics of patients in phase 1

Characteristics	Patients, No (%)<br>Overall (N=9)
Age, median (IQR), y	73.0 (66.0-75.0)
Female sex	5 (55.6)
BMI, median (IQR), kg/m^2	24.3 (22.0-26.0)
**Physician-assigned PROs**	
KCCQ	2 (22.2)
AFEQT	7 (77.8)
SAQ	0 (0)
**Comorbidities**	
Heart failure	3 (33.3)
Atrial fibrillation	8 (88.9)
Coronary artery disease	1 (11.1)
Hypertension	5 (55.6)
Diabetes mellitus	1 (11.1)
Dyslipidemia	5 (55.6)
Chronic kidney disease[a]	5 (55.6)
Stroke	1 (11.1)
Cancer	0 (0)
**Echocardiographic parameters and laboratory tests**	
eGFR, median (IQR), mL/min/1.73 m^2	54.7 (42.7-64.9)
LVEF, median (IQR), %	59.8 (53.0-67.0)
Left atrial diameter, median (IQR), mm	41 (35-43)
BNP, median (IQR), pg/mL	76.4 (42.1-102.0)

Abbreviations: AFEQT, Atrial Fibrillation Effect on Quality-of-Life questionnaire; BNP, B-type natriuretic peptide; BMI, body mass index (calculated as weight in kilograms divided by height in meters squared); eGFR, estimated glomerular filtration rate; KCCQ, Kansas City Cardiomyopathy Questionnaire; PRO, patient-reported outcome; SAQ, Seattle Angina Questionnaire; SD, standard deviation.

[a] Chronic kidney disease was defined as eGFR less than 60 mL/min/1.73 m^2, calculated based on the formula eGFR = 194×serum creatinine (mg/dL)$^{-1.094}$×age$^{-0.287}$×0.739 (if female).

© 2025 Yamashita S et al. *JAMA Network Open.*

eTable 2. Questionnaire to Assess Patient Satisfaction and Quality of Information Provided by Physician

Patient Satisfaction

1. Please choose one of the following options (①-⑤) for each of the following five questions.

① : strongly agree ② : agree ③ : uncertain ④ : disagree ⑤ : strongly disagree

<Question items>	<Subscale>
Q.1 Doctors are good about explaining the reason for medical tests.	Communication
Q.2 When I go for medical care, they are careful to check everything when treating and examining me.	Technical Quality
Q.3 My doctors treat me in a very friendly and courteous manner.	Interpersonal Manner
Q.4 Those who provide my medical care sometimes hurry too much when they treat me.	Time Spent with Doctor
Q.5 Doctors usually spend plenty of time with me.	Time Spent with Doctor

2. Please indicate your satisfaction with the medical care. Using a scale of 0 to 10, where 0 represents the least satisfied and 10 represents the most satisfied.

Quality of Information

1. How would you rate the quality of the information you have been given by your physician about the treatment and the treatment choices available for you?

① : Excellent ② : Good ③ : Satisfactory ④ : Fair ⑤ : Poor

2. How would you rate the quality of the information you have been given by your physician about the likely outcome (i.e. your prognosis) of your disease?

① : Excellent ② : Good ③ : Satisfactory ④ : Fair ⑤ : Poor

© 2025 Yamashita S et al. *JAMA Network Open.*

eTable 3. Questionnaires to Assess Patient Knowledge about Heart Failure and Atrial Fibrillation

A. Heart Failure

Patient knowledge about heart failure
1. Please choose the most typical trajectory of heart failure from the following four figures*.
2. How do you think your heart failure will impact on your life expectancy?
1. HF will finally be cured completely<br>2. HF will last the rest of their lives without shortening their life expectancy<br>3. HF will shorten their life expectancy<br>4. it is unclear how HF would impact their lives.

* The four figures about heart failure trajectory are illustrated in eFigure1.
Abbreviation: HF, heart failure.

B. Atrial Fibrillation

Patient knowledge about atrial fibrillation
1. Why is it important to take my medication for atrial fibrillation properly?
Because the doctor wants me to<br>To prevent severe consequences of the arrhythmia<br>To prevent the possibility of a heart attack or sudden death
2. What is atrial fibrillation?
A heart disease in which the heart is not able to pump a sufficient amount of blood through the body.<br>A blood disorder causing blood clots in the heart.<br>An electronic disorder in the atria of the heart which results in the heart contracting too fast and irregularly.
3. Why is oral anticoagulation medication prescribed in certain patients with atrial fibrillation?

© 2025 Yamashita S et al. *JAMA Network Open.*

To prevent the risk of blood clots which can cause a stroke.
To make the blood flow more easily through the body
To prevent fluid retention in the body

Patient knowledge about atrial fibrillation (continued)

4. Atrial fibrillation is a rare condition

True
False
Don't know.

5. Which statement with regard to physical exercise is true of patients with atrial fibrillation

It is important for patients to rest in order to maintain normal heart activity.
Patients with chronic atrial fibrillation cannot work fulltime.
It is important to exercise normally within personal limitations.

6. Which statement is true?

Atrial fibrillation is endangering because it can result in a heart attack.
Atrial fibrillation is completely harmless.
Atrial fibrillation is harmless if the right medication is taken.

7. Which of the following statements is correct?

If I cough a lot, it is better not to take my heart medication.
If I have no complaints, I can stop taking my heart medication.
It is important that I take my heart medication at fixed times.

8. Patients can detect AF by taking their pulse regularly.

True
False

Abbreviations: AF, atrial fibrillation

© 2025 Yamashita S et al. *JAMA Network Open.*

eTable 4. Qualitative assessment for the prototype ePRO system from patients

Aspect of ePRO system		Exemplar quotes
**Visual presentation**	Facilitator	"My condition is quantified and displayed, making it easy to understand." (Patient 1)
	Suggestion	"It will be easier to understand if the changes in scores before and after are displayed." (Patient 2)<br>"I want to know the meaning of the AFEQT scores. I cannot understand whether a higher or lower score is better." (Patient 9)
**User-friendliness**	Facilitator	"It wasn't a hassle to use the tablet to answer." (Patient 8)
	Suggestion	"It will be good if this system enables us to answer at home, about once a month." (Patient 6)
**Implementation of PRO**	Facilitator	"I think it's good because I can communicate through this system things that I can't discuss with my doctor." (Patient 8)
	Limitation	"It is difficult to respond to questions about symptoms over the past four weeks because they can vary from day to day." (Patient 1)<br>"I have a knee problem and cannot climb stairs; therefore, it is difficult to answer questions about climbing stairs" (Patient 5)

Abbreviations: ePRO, electronic Patient-Reported Outcome; PRO, Patient-Reported Outcome.

© 2025 Yamashita S et al. *JAMA Network Open.*

eTable 5. Qualitative assessment for the prototype ePRO system from physicians

Aspect of ePRO system		Exemplar quotes
**Visual presentation**	Suggestion	"Visual representation is easy to understand and helpful. It would be great if the domains related to the PRO summary score could be visually represented." (Physician 1)
	Suggestion	"I think line graphs or bar charts are effective for representing PRO trajectories. It would be better if the representation is easy for patients to understand." (Physician 2)
**User-friendliness**	Facilitator	"I don't think the system's operation is particularly difficult." (Physician 2)
**Implementation of PRO**	Facilitator	"I think the time burden associated with ePRO monitoring will be a key point in implementing this system." (Physician 1)

Abbreviations: ePRO, electronic Patient-Reported Outcome; PRO, Patient-Reported Outcome.

© 2025 Yamashita S et al. *JAMA Network Open.*

eTable 6. Changes in Patient Satisfaction and Quality of Information Provided by Physician

	Baseline	Follow-up	Absolute change	
	Mean (SD)	Mean (SD)	Mean (SD)	*P-value*
PSQ score				
control (n=25)	22.00 (2.38)	22.16 (2.21)	0.16 (2.06)	0.012
ePRO (n=23)	20.65 (2.39)	22.26 (2.54)	1.61 (1.75)	
Satisfaction (Likert scale)				
control (n=25)	8.52 (1.73)	8.16 (2.36)	-0.36 (2.53)	0.21
ePRO (n=23)	8.65 (1.64)	9.00 (1.21)	0.35 (0.88)	
Quality of information about treatment				
control (n=25)	4.28 (0.61)	4.16 (0.62)	-0.12 (0.53)	0.013
ePRO (n=23)	3.83 (0.94)	4.17 (0.72)	0.35 (0.71)	
Quality of Information about prognosis				
control (n=25)	4.32 (0.48)	4.08 (0.70)	-0.24 (0.52)	0.08
ePRO (n=23)	3.96 (0.71)	4.04 (0.82)	0.09 (0.73)	

Values are mean (SD).

Abbreviations: ePRO, electronic Patient-Reported Outcome; PSQ, Patient Satisfaction Questionnaire.

© 2025 Yamashita S et al. *JAMA Network Open.*

eTable 7. Changes in Individual Components of the 5-items of Patient Satisfaction Questionnaire

		Absolute change		
<Question items>	<Subscale>	Control (N=25)	ePRO (N=23)	*P-value*
Q.1 Doctors are good about explaining the reason for medical tests.	Communication	-0.12 (0.53)	0.43 (0.90)	0.011
Q.2 When I go for medical care, they are careful to check everything when treating and examining me.	Technical Quality	-0.08 (0.49)	0.22 (0.60)	0.066
Q3. My doctors treat me in a very friendly and courteous manner.	Interpersonal Manner	0.20 (0.50)	0.17 (0.49)	0.856
Q4. Those who provide my medical care sometimes hurry too much when they treat me.	Time spent with doctor	0.04 (0.84)	0.48 (0.73)	0.061
Q5. Doctors usually spend plenty of time with me.	Time spent with doctor	0.12 (0.60)	0.30 (0.82)	0.377

Abbreviations: ePRO, electronic Patient-Reported Outcome.

© 2025 Yamashita S et al. *JAMA Network Open.*

eTable 8. PRO scores at baseline and last follow-up in the ePRO group

	Baseline<br>Mean (SD)	Last follow-up<br>Mean (SD)	Absolute change<br>Mean (SD)	*P-value**
**KCCQ (N=11)**				
Physical limitation	68.9 (25.3)	67.5 (25.0)	1.7 (21.8)	0.81
Symptom frequency	73.3 (28.3)	70.1 (25.1)	-3.2 (25.3)	0.68
Quality of life	47.7 (20.8)	51.1 (32.3)	3.4 (32.2)	0.73
Social limitation	52.5 (28.7)	58.3 (39.1)	14.8 (26.7)	0.13
KCCQ clinical summary score	61.1 (21.7)	63.5 (28.1)	2.3 (19.5)	0.70
KCCQ summary score	71.1 (23.5)	70.3 (23.9)	-0.85 (20.3)	0.89
**AFEQT (N=10)**				
Symptom	62.9 (26.4)	83.3 (12.9)	20.4 (23.1)	0.021
Daily activities	59.2 (15.9)	75.6 (10.6)	16.5 (20.9)	0.035
Treatment concern	68.9 (13.5)	83.3 (9.0)	14.4 (11.6)	0.0034
Satisfaction	65.0 (18.8)	70.8 (18.5)	5.8 (20.1)	0.38
AFEQT overall summary score	63.2 (12.2)	79.9 (8.4)	16.7 (14.2)	0.0047
**SAQ (N=2)**				
SAQ summary score	85.0 (7.9)	92.6 (1.8)	7.6 (6.2)	0.33

Abbreviations: AFEQT, Atrial Fibrillation Effect on Quality-of-Life questionnaire; ePRO, electronic patient-reported outcome; KCCQ, Kansas City Cardiomyopathy Questionnaire; PRO, patient-reported outcome; SAQ, Seattle Angina Questionnaire; SD, standard deviation.

*P-values were calculated by comparing the PRO scores at baseline and last follow-up for KCCQ, AFEQT and SAQ using a paired t-test.

© 2025 Yamashita S et al. *JAMA Network Open.*

eFigure 1. Evolution of the electronic patient-reported outcome survey system

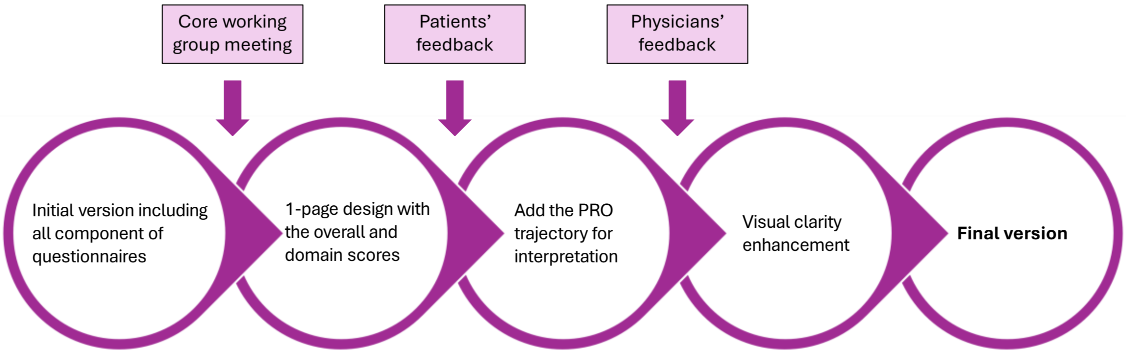

Abbreviations: PRO, patient-reported outcome; KCCQ-12, the Kansas City Cardiomyopathy Questionnaire-12.

© 2025 Yamashita S et al. *JAMA Network Open.*

eFigure 2. Workflow of electronic patient-reported outcome monitoring

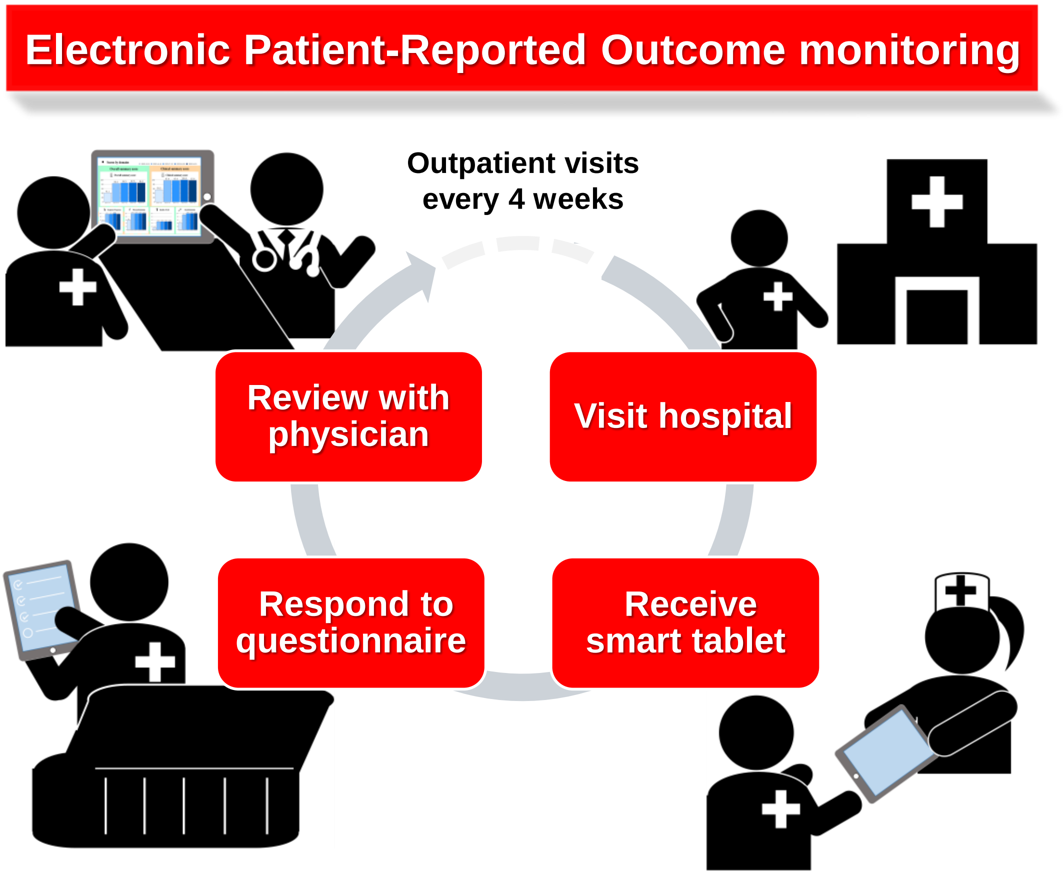

Abbreviations: eRO, electronic patient-reported outcome.

© 2025 Yamashita S et al. *JAMA Network Open.*

eFigure 3. Four figures representing trajectories of various illnesses

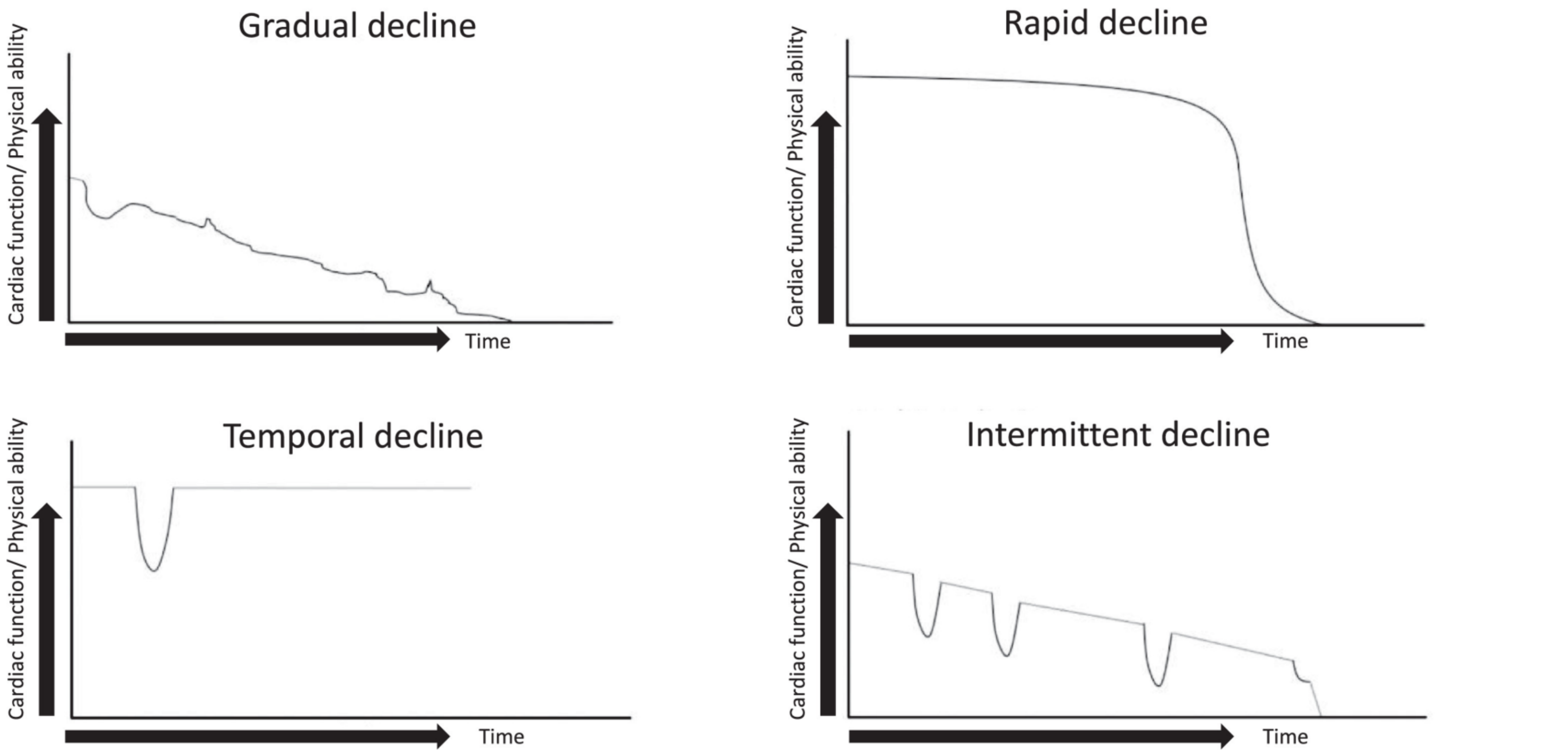

To evaluate patient knowledge about heart failure trajectory, patients with heart failure were instructed to choose the most typical trajectory of HF from among the four figures.

© 2025 Yamashita S et al. *JAMA Network Open.*

eFigure 4. Patients' assessment of the prototype ePRO system regarding visualization and survey method

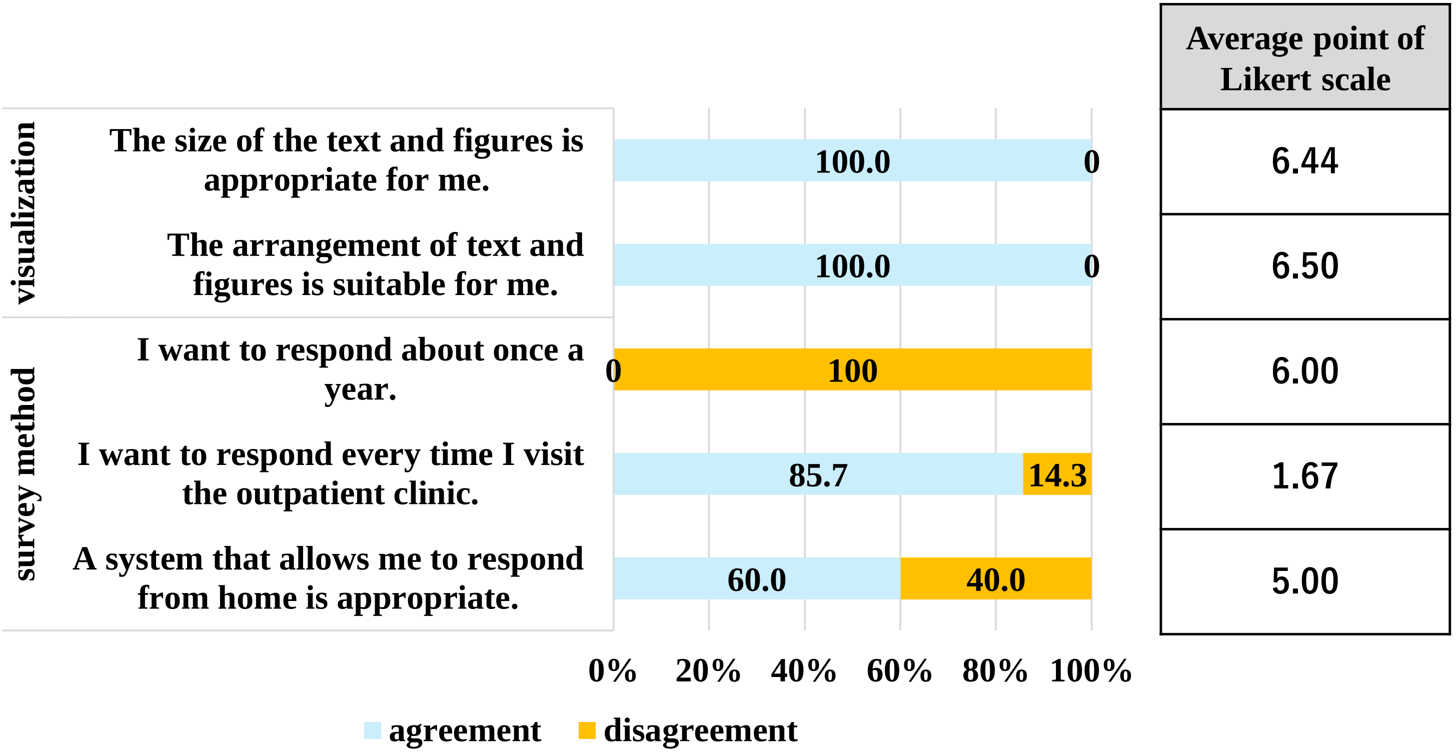

Patients responded to each question using a seven-point Likert scale (1: strongly disagree, 2: disagree, 3: somewhat disagree, 4: neither agree nor disagree, 5: somewhat agree, 6: agree, 7: strongly agree). The proportion of responses indicating agreement (5: somewhat agree, 6: agree, 7: strongly agree) or disagreement (1: strongly disagree, 2: disagree, 3: somewhat disagree)

© 2025 Yamashita S et al. *JAMA Network Open.*

was represented, as no participants selected 'neither agree nor disagree' for each question. Average point of a seven-point Likert scale was shown at the right side of the bar graph. Abbreviations: ePRO, electronic Patient-Reported Outcome.

© 2025 Yamashita S et al. *JAMA Network Open.*

eFigure 5. Example of KCCQ report displayed on a screen

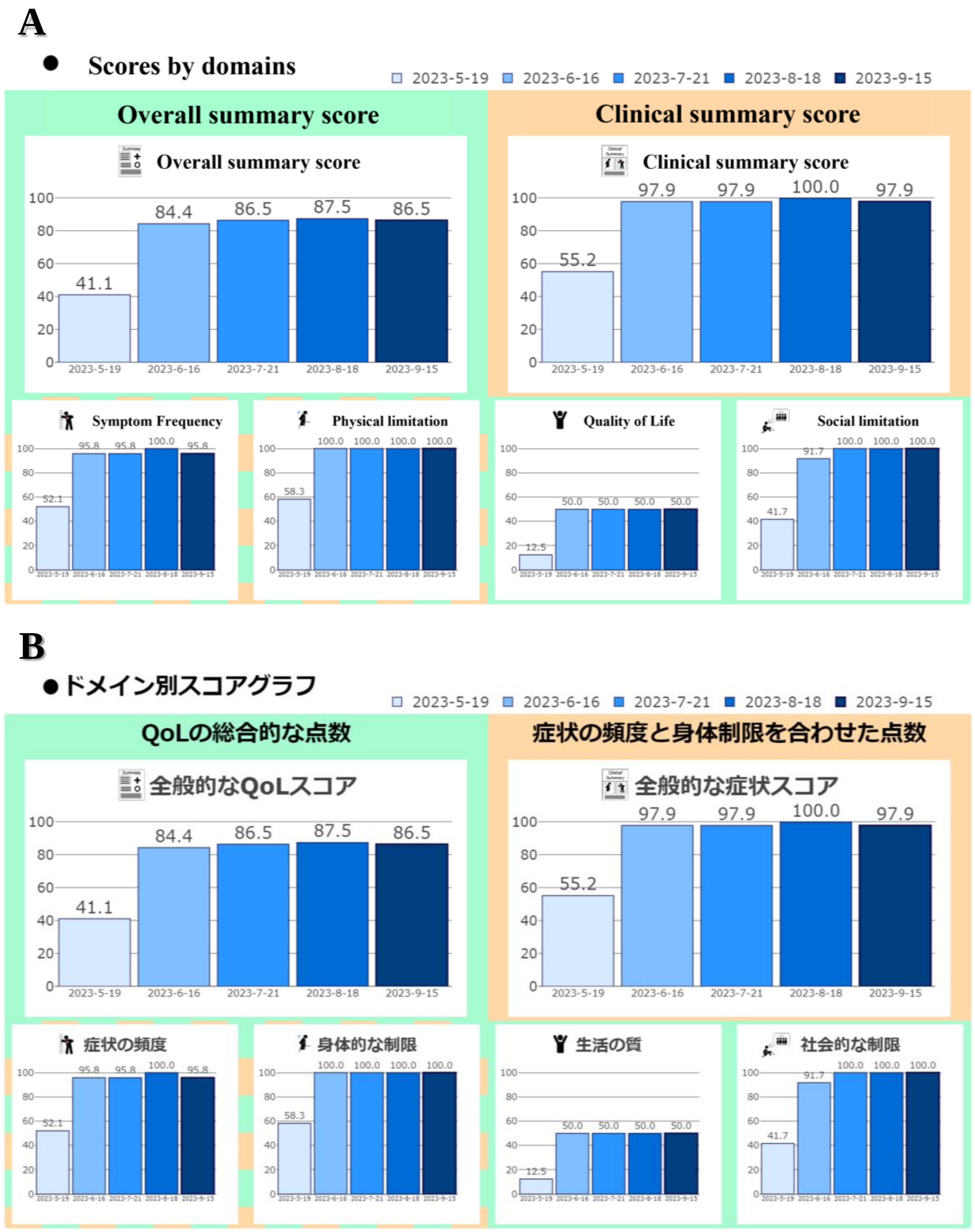

Example of KCCQ-12 report with English annotations (A) and original Japanese version (B).

Abbreviations: KCCQ-12, the Kansas City Cardiomyopathy Questionnaire-12.

© 2025 Yamashita S et al. *JAMA Network Open.*

eFigure 6. Example of AFEQT report displayed on a screen

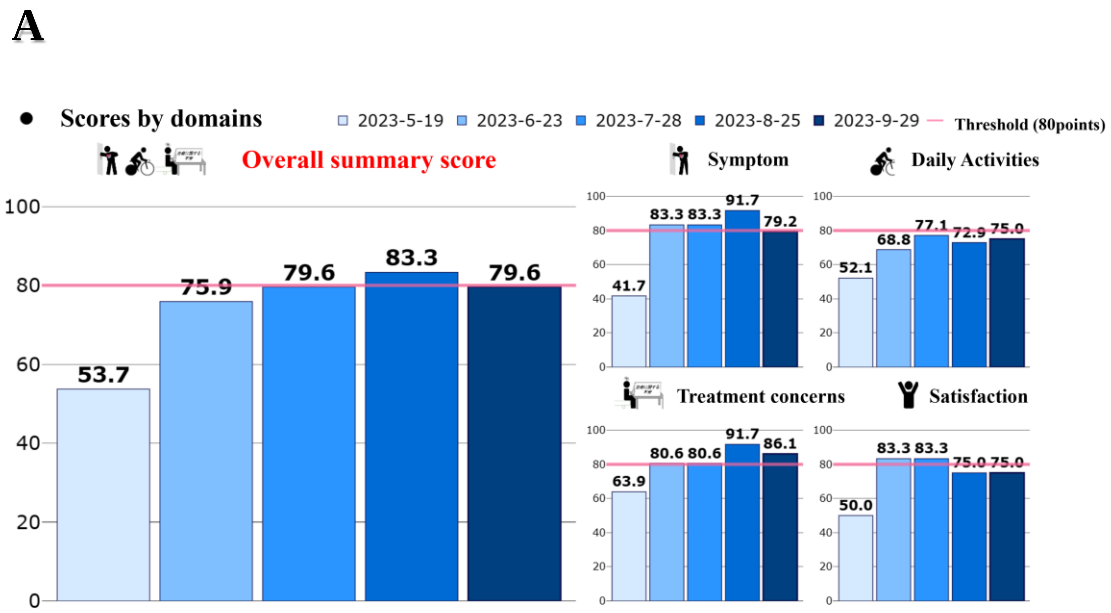

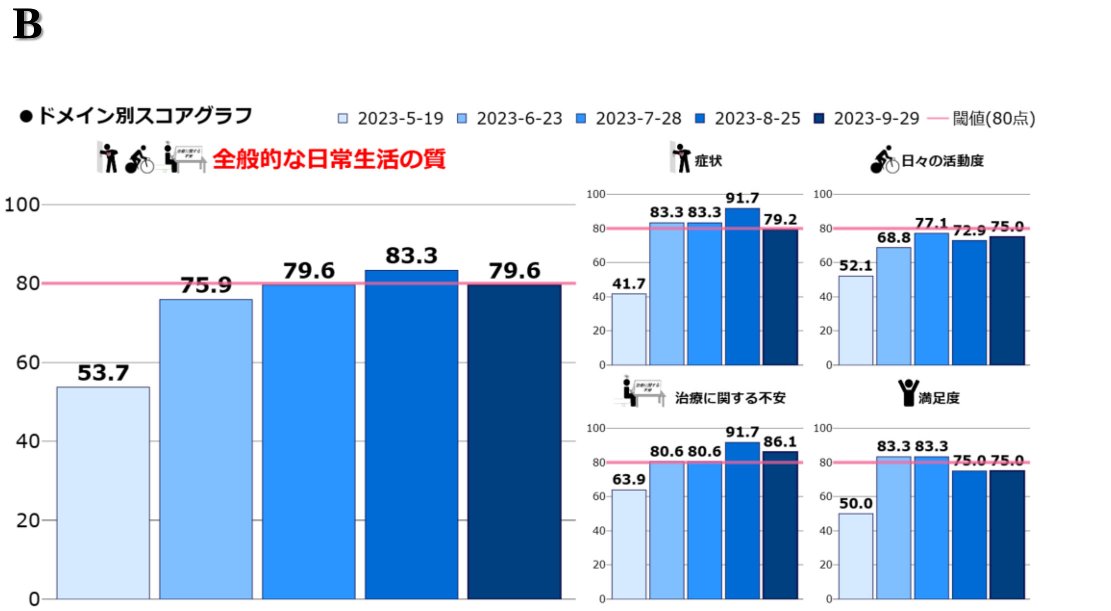

Example of AFEQT report with English annotations (A) and original Japanese version (B).

Abbreviations: AFEQT, the AF Effect on Quality of Life questionnaire.

© 2025 Yamashita S et al. *JAMA Network Open.*

eFigure 7. Example of SAQ report displayed on a screen

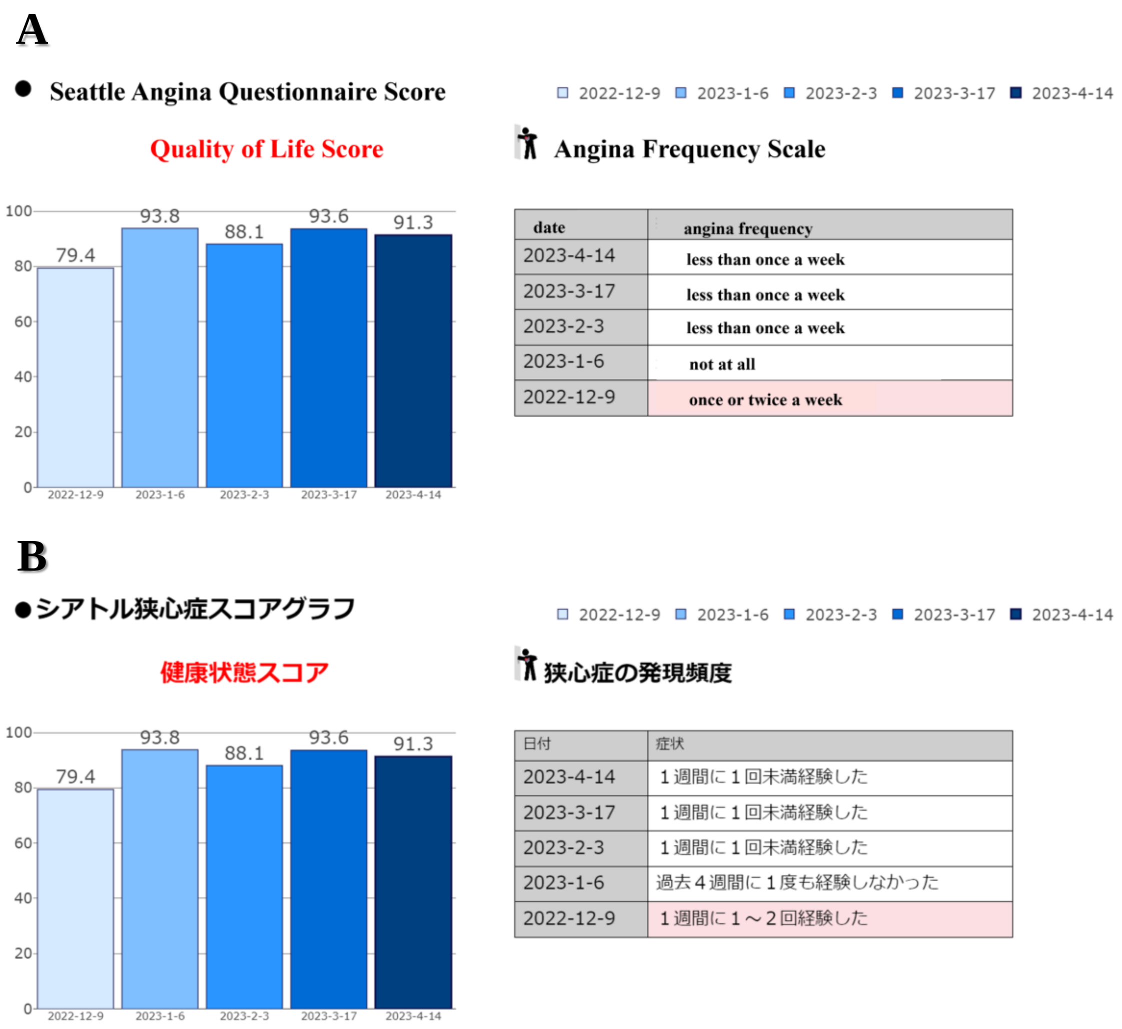

Example of SAQ report with English annotations (A) and original Japanese version (B).

Abbreviations: SAQ, the Seattle Angina Questionnaire.

© 2025 Yamashita S et al. *JAMA Network Open.*
