# Supplementary material for: Electronic Patient-Reported Outcome System Implementation in Outpatient Cardiovascular Care: A Randomized Clinical Trial
Source: JAMA Netw Open. 2025 Jan 14;8(1):e2454084. doi: 10.1001/jamanetworkopen.2024.54084 (PMC11733702; doi:10.1001/jamanetworkopen.2024.54084)
Supplement: Supplement 3. — Data Sharing Statement [file jamanetwopen-e2454084-s003.pdf]

Data Sharing Statement

Yamashita. Electronic Patient-Reported Outcome System Implementation in Outpatient Cardiovascular Care. *JAMA Netw Open*. Published January 14, 2025. doi:10.1001/jamanetworkopen.2024.54084

Data
Additional Information: Name of the trial registry: Study of usefulness of ePRO in cardiovascular disease Registry's URL: umin.ac.jp/ctr Trial registration number: UMIN000049251
Data available: No

Additional Information
Explanation for why data not available: The data and materials used to conduct this research are available to researchers, for scientific projects aims at identifying a novel clinical finding that may further improve patient care and outcomes.
